# Interventions to Reduce Mental Health Stigma in Young People: A Systematic Review and Meta-Analysis

**DOI:** 10.1001/jamanetworkopen.2024.54730

**Published:** 2025-01-15

**Authors:** Marcelo A. Crockett, Daniel Núñez, Pablo Martínez, Francesca Borghero, Susana Campos, Álvaro I. Langer, Jimena Carrasco, Vania Martínez

**Affiliations:** 1 Millennium Nucleus to Improve the Mental Health of Adolescents and Youths (IMHAY), Santiago, Chile; 2 Associative Research Program, Research Center on Cognitive Sciences, Facultad de Psicología, Universidad de Talca, Talca, Chile; 3 McGill Group for Suicide Studies, Douglas Mental Health University Institute, Department of Psychiatry, McGill University, Montreal, Quebec, Canada; 4 Millennium Institute for Research in Depression and Personality (MIDAP), Santiago, Chile; 5 Center of Applied Psychology, Faculty of Psychology, Universidad de Talca, Talca, Chile; 6 Facultad de Psicología y Humanidades, Universidad San Sebastián, Valdivia, Chile; 7 Facultad de Medicina, Universidad Austral de Chile, Valdivia, Chile; 8 Centro de Medicina Reproductiva y Desarrollo Integral del Adolescente (CEMERA), Facultad de Medicina, Universidad de Chile, Santiago, Chile

## Abstract

**Importance:**

Mental health stigma is a considerable barrier to help-seeking among young people.

**Objective:**

To systematically review and meta-analyze randomized clinical trials (RCTs) of interventions aimed at reducing mental health stigma in young people.

**Data Sources:**

Comprehensive searches were conducted in the CENTRAL, CINAHL, Embase, PubMed, and PsycINFO databases from inception to February 27, 2024. Search terms included “stigma,” “mental health,” “mental disorders,” “adolescents,” “youth,” and “randomized controlled trial.”

**Study Selection:**

Inclusion criteria encompassed RCTs involving interventions aimed at reducing mental health stigma among young people (aged 10-24 years). Studies had to report outcomes related to stigma or help-seeking behaviors. Exclusion criteria included grey literature and studies without results.

**Data Extraction and Synthesis:**

Data were extracted independently by 7 authors (M.A.C., D.N., F.B., S.C., Á.I.L., J.C., V.M.) using Preferred Reporting Items for Systematic Reviews and Meta-Analyses guidelines. Risk of bias was assessed with the Cochrane risk-of-bias tool. Three-level multivariate meta-analyses were conducted to account for within-study correlations and to maximize data use. Standardized mean differences (SMDs) (Hedges *g*) and odds ratios (ORs) with 95% CIs were calculated. The data analysis was conducted from May 30 through July 4, 2024.

**Main Outcomes and Measures:**

Primary outcomes included stigma-related knowledge, attitudes, behaviors, and general stigma. Help-seeking outcomes were categorized into attitudes, intentions, and behaviors. Secondary outcomes included self-efficacy and empowerment.

**Results:**

A total of 97 studies were included in the systematic review, representing 43 852 young people (mean [IQR] age, 18.7 [15.8-21.3] years; mean [IQR] females, 59.2% [49.4%-72.0%]), and 74 studies were included in 3-level multivariate meta-analyses. Significant short-term effect sizes were found for stigma-related knowledge (SMD, 0.66; 95% CI, 0.43-0.89), attitudes (SMD, 0.38; 95% CI, 0.20-0.56), behaviors (SMD, 0.29; 95% CI, 0.13-0.45), and general stigma (SMD, 0.20; 95% CI, 0.06-0.34) and for help-seeking attitudes (SMD, 0.18; 95% CI, 0.09-0.28) and intentions (SMD, 0.14; 95% CI, 0.07-0.21). Social contact interventions had a greater influence on stigma-related behaviors than did educational approaches.

**Conclusions and Relevance:**

These findings suggest that interventions to reduce mental health stigma among youth are beneficial in the short term. Further high-quality RCTs with long-term follow-up are needed to better understand and enhance these interventions’ outcomes.

## Key Points

**Question:**

Are interventions aimed at reducing mental health stigma in young people associated with improved outcomes?

**Findings:**

In this systematic review and meta-analysis of 97 randomized clinical trials involving 43 852 young people (aged 10-24 years), interventions had a significant short-term association with improvements in stigma-related knowledge, attitudes, and behaviors and help-seeking attitudes and intentions.

**Meaning:**

These findings suggest that interventions to reduce mental health stigma may be effective in the short term, but further high-quality studies are needed to assess long-term outcomes.

## Teaser

This systematic review and meta-analysis of randomized clinical trials examined whether interventions aimed at reducing mental health stigma are associated with improved outcomes in young people.

## Introduction

Stereotypes, prejudice, or discrimination against individuals with mental illness, known as mental health stigma,^1^ present considerable barriers to help-seeking among young people with mental health conditions^2,3^ (ie, mental disorders; psychosocial disabilities; other mental states associated with distress, impairment, or self-harm risk^4^). This stigma affects individuals and communities across cultures and is linked to several negative consequences in personal, structural, health, social, and economic domains.^5^ Reducing mental health stigma is crucial, especially in youth, as untreated mental illness can have lasting effects.^6,7,8^ Educational approaches and social contact with individuals with mental illness are commonly studied interventions to reduce stigma among young people.^5,9^ These interventions have been shown to positively influence stigma-related attitudes and knowledge in the short term, with educational approaches being more effective in school-age populations^10,11,12,13^ and social contact interventions being more effective in higher education students.^14,15^ However, more high-quality studies with more extended follow-up periods are needed to understand the lasting impact of these interventions.^5,9^ Previous meta-analyses on stigma interventions have not accounted for the correlation between mental health stigma measures within the same participants across studies.^1,16,17^ Addressing this correlation may ensure accurate estimation of each outcome without conflation.^18,19^ To fill this gap, we conducted a systematic review of randomized clinical trials (RCTs) on interventions to reduce mental health stigma in young people, using 3-level multivariate meta-analyses to account for these correlations and maximize data use.^18,19^ In addition, differences in the effect size of interventions based on study characteristics were examined.

## Methods

This systematic review and meta-analysis followed the Preferred Reporting Items for Systematic Review and Meta-Analysis (PRISMA) guideline. The study protocol was preregistered with PROSPERO (CRD42020210901) and published.^20^ Protocol amendments are documented in eAppendix 1 in Supplement 1. Human research ethics committee approval and informed consent were not required per the Agencia Nacional de Investigación y Desarrollo ethics guidelines because anonymized information from publicly available studies was analyzed.

### Eligibility Criteria

We searched studies in peer-reviewed journals indexed in the CENTRAL, CINAHL, Embase, PubMed, and PsycINFO databases from inception until February 27, 2024. Articles in English and Spanish were included to expand the search for articles from different contexts. We excluded grey literature, study protocols, and reports without results.

The study eligibility criteria followed the population, interventions, comparators, and outcomes framework. The population included young people aged 10 to 24 years with or without mental health problems. Interventions included those aimed at reducing mental health stigma, including educational approaches (ie, conferences, text reading, role play), social contact, famous films, or other types.^15^ Comparators included active comparison (antistigma intervention), standard treatment, waiting list, and placebo or no intervention.

Outcomes were based on a sociocognitive model of stigma and a framework for help-seeking for mental health problems.^1,21^ The primary outcomes were stigma-related knowledge, attitudes, behaviors, and general stigma (outcomes that measure knowledge, attitudes, and/or behaviors at the same time) and help-seeking attitudes, intentions, and behaviors further divided into formal (eg, health professionals) and informal (eg, friends) sources. Secondary outcomes included helping attitudes and behaviors, self-efficacy, and empowerment. Timing was categorized as short term (<3 months’ follow-up), midterm (3-6 months’ follow-up), and long term (>6 months’ follow-up).

Study designs included RCTs and controlled clinical trials (ie, where randomization was not explicitly reported but cannot be ruled out). Systematic reviews and meta-analyses were examined for eligible primary studies.

### Search Strategy

The detailed search strategy is included in eAppendix 2 in Supplement 1. The basic search strategy combined index and free-text terms for “stigma” AND (“mental health” OR “mental disorders”) AND (“adolescents” OR “youth”) AND “randomized controlled trial.”

### Study Selection and Data Collection Process

Study selection and data extraction were conducted by 7 authors independently (M.A.C., D.N., F.B., S.C., Á.I.L., J.C., V.M.) and in duplicate until high agreement was reached. The authors then reviewed the remaining studies independently. The results were discussed among the reviewers.

Data were extracted between May 14, 2021, and April 26, 2022, and updated between February 27 and May 28, 2024. Data extracted included the following: (1) study identification (authors, year of publication, and country), (2) characteristics of the study sample and methods (age range, gender, presence and type of mental illness, criteria used to define stigma, study design, recruitment methods, and inclusion and exclusion criteria), (3) characteristics of interventions and comparators (periodicity, duration, format, content, target population, and type of stigma addressed), (4) characteristics of outcomes (type of outcome measure, measurement instruments, and timing), and (5) study results (means and standard deviations for continuous outcomes or number of events and total number of participants for dichotomous outcomes per each group).

### Risk of Bias in Individual Studies

The Cochrane risk-of-bias tool was used to assess the risk of selection; performance; detection; attrition; and notification bias, classified as high, low, or unclear.^22^ This assessment followed the same procedures as study selection and data collection.

### Statistical Analysis

To account for the interdependence of stigma-related outcomes, we performed a 3-level multivariate meta-analysis, modeling within-study correlations to improve precision and reduce bias.^18,19^ This approach allowed us to borrow strength across related outcomes, enhancing the robustness of our effect size estimates compared with univariable analyses.^18,19^ For the meta-analysis, all articles that provided sufficient data for primary and/or secondary outcomes were included. If basic summary measures were not reported (eg, mean and standard deviation), we followed the Cochrane Collaboration’s recommended procedures.^23^ Meta-analyses considered the timing of outcome measurement, the type of outcomes, and the type of comparison as stratification variables.

Effect sizes were the standardized mean difference (SMD) (Hedges *g*) and odds ratio (OR) for continuous and dichotomous outcomes, respectively, with 95% CIs. Unique identifiers for each effect size and study were included as random effects to account for data structure. We approximated the variance-covariance matrix of dependent within-study effect sizes by assuming a strong correlation (0.75) within outcome categories or a moderate correlation (0.40) otherwise.^16^ These meta-analyses estimated between- and within-study variance components with heterogeneity statistics (*I*^2^).^16^ Forest plots were used to visually display the results of meta-analyses that included at least 10 studies.

Moderation analyses were performed using meta-regression techniques in meta-analyses of at least 10 studies to ensure statistical power,^24^ with the aim of examining possible differences in the effect size of the interventions based on the characteristics of the studies. Univariable meta-regressions were used to evaluate selected study characteristics (ie, risk-of-bias assessment, type of intervention, intervention format, sample age).

Publication bias was assessed in meta-analyses of at least 10 studies through funnel plots and univariable 3-level meta-regression, with the standard error of effect sizes as the independent variable. All analyses were performed using the metafor package in R, version 4.2.0 (R Foundation).^25^ The data analysis was conducted from May 30 through July 4, 2024.

## Results

### Study Selection and Characteristics

**Figure 1.**
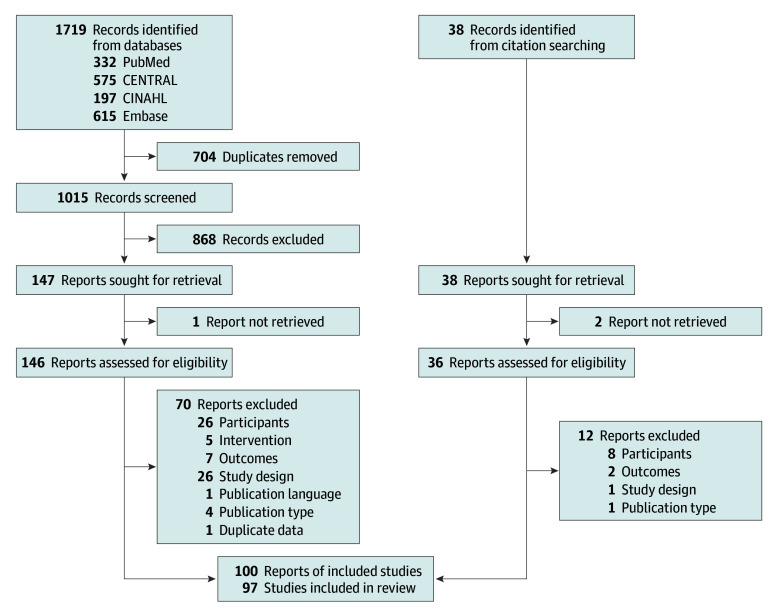
Flow Diagram of the Study Selection Process

The study selection process concluded with the inclusion of 97 studies^26,27,28,29,30,31,32,33,34,35,36,37,38,39,40,41,42,43,44,45,46,47,48,49,50,51,52,53,54,55,56,57,58,59,60,61,62,63,64,65,66,67,68,69,70,71,72,73,74,75,76,77,78,79,80,81,82,83,84,85,86,87,88,89,90,91,92,93,94,95,96,97,98,99,100,101,102,103,104,105,106,107,108,109,110,111,112,113,114,115,116,117,118,119,120,121,122,123,124,125^ for the systematic review and 74 studies^51,52,53,54,55,56,57,58,59,60,61,62,63,64,65,66,67,68,69,70,71,72,73,74,75,76,77,78,79,80,81,82,83,84,85,86,87,88,89,90,91,92,93,94,95,96,97,98,99,100,101,102,103,104,105,106,107,108,109,110,111,112,113,114,115,116,117,118,119,120,121,122,123,124,125^ for the 3-level multivariate meta-analyses (Figure 1). Most studies were individual-level RCTs (62 studies [63.9%])^26,27,31,32,33,37,38,39,40,42,43,45,46,47,48,50,51,52,53,54,55,56,57,58,59,61,62,63,65,66,68,69,71,73,74,75,79,82,84,86,87,89,94,95,96,97,98,99,100,102,103,106,107,108,110,112,115,116,117,119,121,122^ and came from developed countries, mainly the US (30 studies [30.9%]).^26,27,33,39,41,47,48,51,56,58,59,62,71,76,79,82,83,84,95,96,97,98,99,100,106,112,113,115,122,123^ The included studies represented 43 852 young people (mean [range], 452 [32-6679] individuals; mean [IQR] age, 18.7 [15.8-21.3] years; mean [IQR] gender, 59.2% [49.4%-72.0%] female, 40.0% [27.7%-50.0%] male, and 0.8% [0.0%-0.0%] transgender or nonbinary or other gender) and frequently comprised college students (45 studies [46.4%]).^27,30,32,33,37,38,39,40,41,46,47,50,51,54,55,56,57,58,59,61,62,63,65,68,71,76,79,82,84,86,87,89,90,94,95,96,97,106,107,112,118,119,120,121,122^ A total of 28 studies [28.9%] reported results of mixed interventions, ^28,32,40,41,57,63,65,66,72,77,78,80,83,86,88,90,92,94,101,104,105,106,118,119,120,121,123,125^ with social contact plus conferences (ie, a type of educational approach) being the most studied combination (7 studies [7.2%]).^28,57,83,86,90,101,119^ For single interventions, educational conferences were the most studied type of intervention (16 studies [16.5%]).^29,30,36,59,67,74,76,81,85,87,96,97,111,113,114,116^ A total of 91 studies (93.8%) assessed stigma-related outcomes, ^26,27,28,29,30,31,32,33,34,35,36,37,38,40,41,42,43,44,45,46,47,48,50,51,52,53,54,55,56,57,58,59,60,64,65,66,67,68,69,71,72,73,74,75,76,77,78,79,80,81,82,83,84,85,86,87,88,89,90,91,92,93,94,95,96,97,98,99,100,101,102,103,104,105,106,107,108,110,111,112,113,115,116,117,118,119,120,121,123,124,125^ 39 (40.2%) provided data for help-seeking outcomes, ^27,32,37,39,46,50,57,58,61,62,63,66,67,69,72,75,76,88,95,96,97,99,100,101,102,103,104,108,110,111,112,113,114,115,116,120,122,124,125^ and 11 (11.3%) provided secondary outcomes. ^42,51,65,81,88,94,102,106,114,116,124^ Further details are presented in eTable 1 in Supplement 1.

### Risk of Bias Within Studies

Studies were most frequently assessed with a high risk of bias on masking of participants and personnel (59 studies [60.8%])^27,28,29,30,31,32,33,34,38,40,42,43,45,47,48,50,54,55,56,59,60,63,64,66,67,68,69,74,76,77,78,80,81,82,83,84,85,86,87,88,89,90,92,93,94,96,97,102,104,105,106,107,111,113,114,116,117,119,125^ and masking of outcome assessment (43 studies [44.3%]),^27,28,29,30,34,38,39,40,41,42,45,47,56,59,60,61,64,67,74,76,77,78,79,80,81,82,84,85,87,89,90,92,93,94,97,105,106,107,111,114,118,123,125^ with an unclear risk of bias on allocation concealment (50 studies [51.5%]).^26,27,29,30,32,33,34,35,36,39,40,45,46,47,52,54,55,56,59,61,62,64,67,71,73,74,76,78,79,81,82,84,85,87,93,95,96,97,98,99,100,105,106,107,113,115,117,118,123,124^ A low risk of bias was commonly found for selective outcome reporting (83 studies [85.6%]),^27,28,29,32,33,36,37,38,39,40,43,44,48,50,51,52,53,54,55,56,57,58,59,60,61,62,64,65,66,68,69,71,72,73,74,75,76,77,78,79,80,81,82,83,84,85,86,87,88,89,90,91,92,93,94,95,96,97,98,99,100,102,103,104,105,106,107,108,110,111,112,114,115,116,117,118,119,120,121,122,123,124,125^ random sequence generation (68 studies [70.1%]),^28,29,30,32,33,34,35,37,38,39,41,42,45,46,47,50,51,53,54,55,58,59,60,61,62,63,64,65,67,68,69,71,72,74,75,76,77,78,79,82,85,88,89,90,91,92,93,94,97,101,102,103,104,105,106,107,108,110,111,112,113,114,116,117,120,121,122,123^ and incomplete outcome data (57 studies [58.8%])^31,32,34,36,37,40,42,43,44,45,46,50,51,52,53,54,55,56,57,62,63,65,66,68,69,71,72,76,78,79,83,84,86,87,88,89,90,92,93,94,95,97,101,102,103,108,110,112,114,115,116,117,120,121,122,123,124^ (eTable 2 in Supplement 1).

### Syntheses of Results

#### Outcomes Associated With Stigma

**Figure 2.**
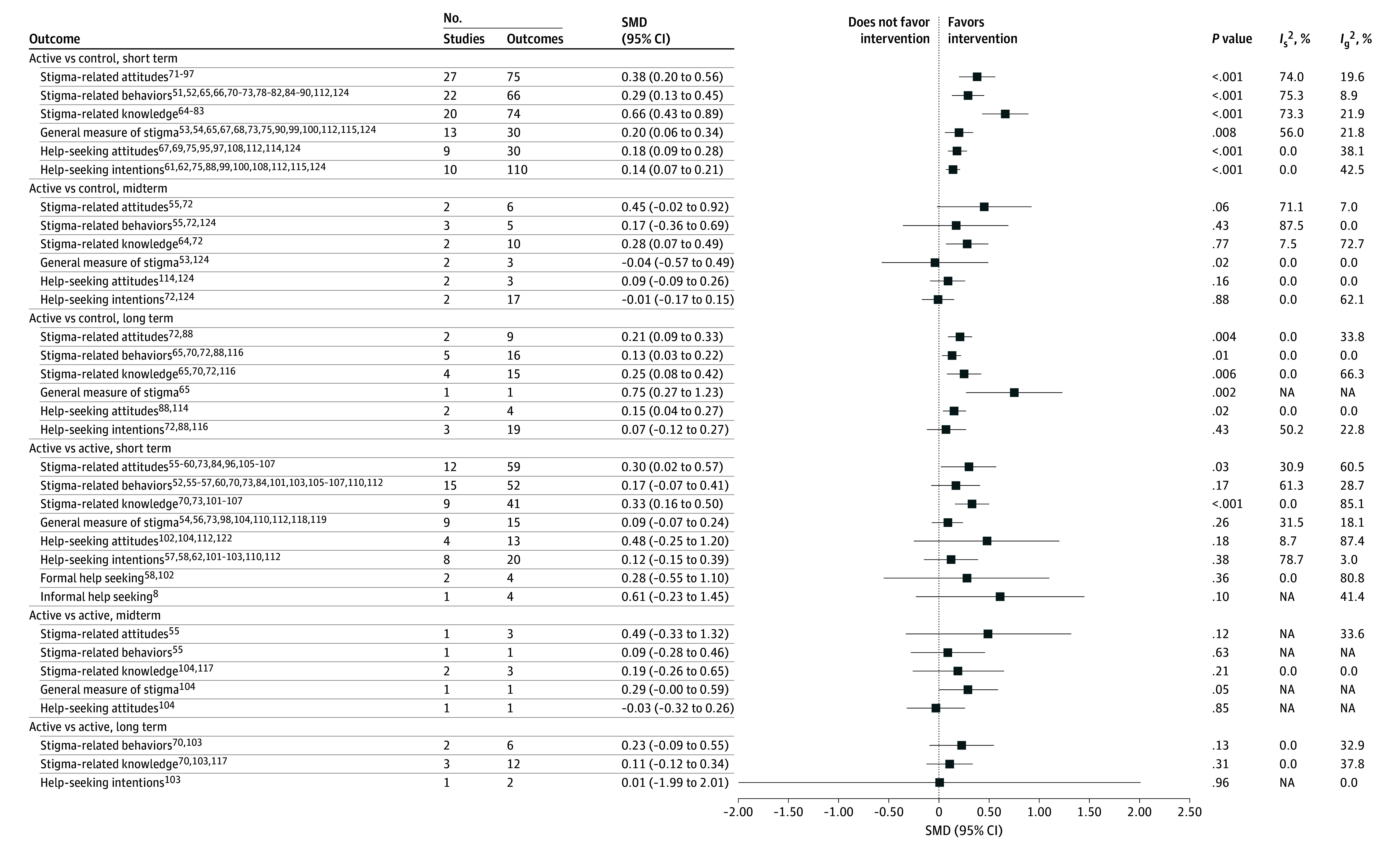
Association of Interventions With Reduced Mental Health Stigma in Young People (Continuous Outcomes) Estimates are from 3-level multivariate meta-analyses. *I*_g_^2^ indicates within-study heterogeneity; *I*_s_^2^, between-study heterogeneity; NA, not applicable; SMD, standardized mean difference.

**Figure 3.**
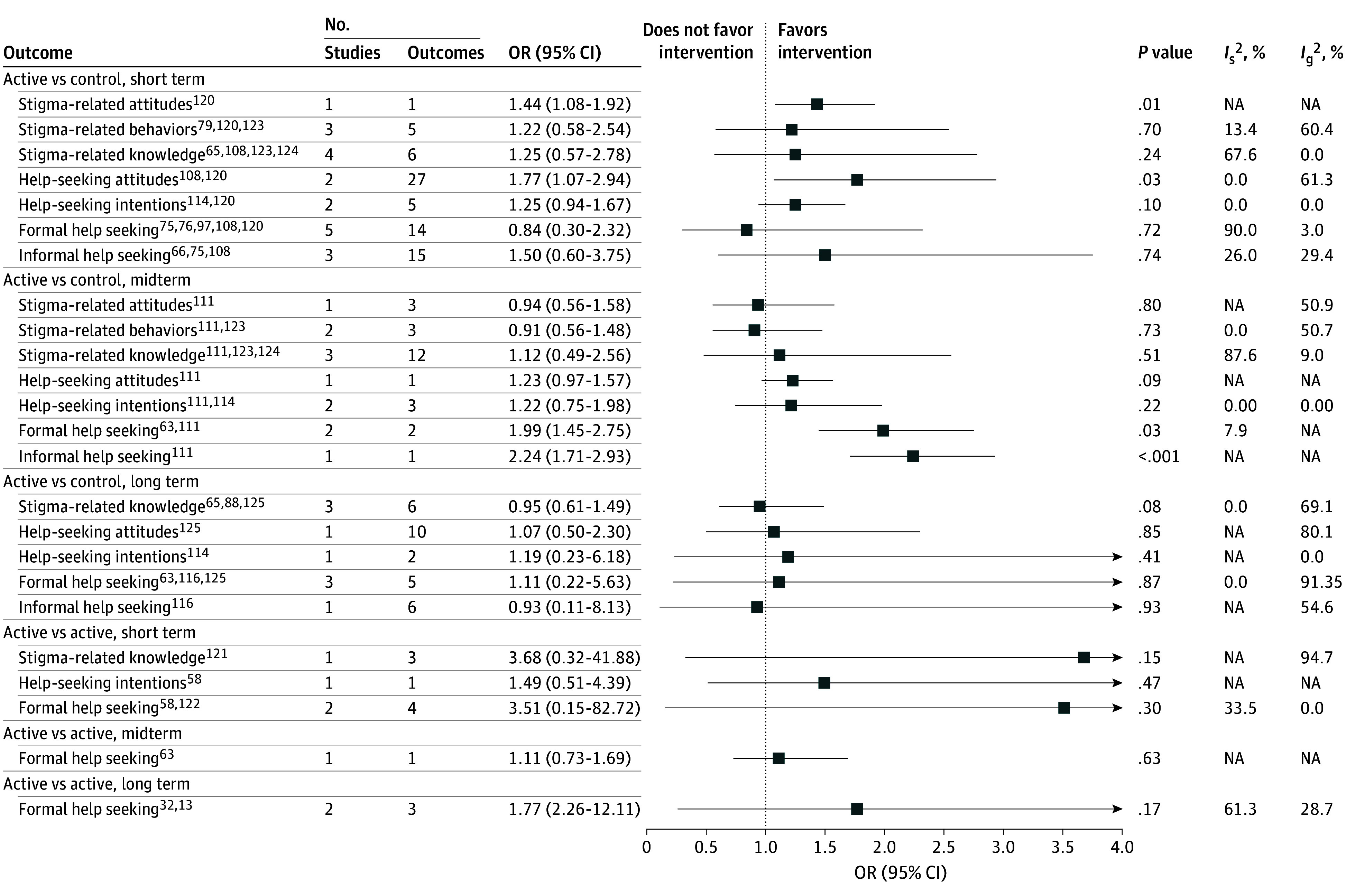
Association of Interventions With Reduced Mental Health Stigma in Young People (Dichotomous Outcomes) Estimates from 3-level multivariate meta-analyses. *I*_g_^2^ indicates within-study heterogeneity; *I*_s_^2^, between-study heterogeneity; NA, not applicable; OR, odds ratio.

As shown in Figure 2, meta-analyses of continuous data for short-term effects of active vs control interventions revealed a significant medium effect size for knowledge (SMD, 0.66; 95% CI, 0.43-0.89; 20 studies; *I*^2^ = 73.3%)^64,65,66,67,68,69,70,71,72,73,74,75,76,77,78,79,80,81,82,83^ and significant small effect sizes for attitudes (SMD, 0.38; 95% CI, 0.20-0.56; 27 studies; *I*^2^ = 74.0%),^71,72,73,74,75,76,77,78,79,80,81,82,83,84,85,86,87,88,89,90,91,92,93,94,95,96,97^ behaviors (SMD, 0.29; 95% CI, 0.13-0.45; 22 studies; *I*^2^ = 75.3%),^51,52,65,66,70,71,72,73,78,79,80,81,82,84,85,86,87,88,89,90,112,124^ and general stigma (SMD, 0.20; 95% CI, 0.06-0.34; 13 studies; *I*^2^ = 56.0%).^53,54,65,67,68,73,75,90,99,100,112,115,124^ A single study reporting dichotomous data showed a significant short-term effect on attitudes (OR, 1.44; 95% CI, 1.08-1.92)^120^ (Figure 3).

For midterm effects of active vs control interventions (Figure 2), there was a significant small effect size for knowledge (SMD, 0.28; 95% CI, 0.07-0.49; 2 studies; *I*^2^ = 7.5%).^64,72^ For long-term effects, a single study showed a significant medium effect size for general stigma (SMD, 0.75; 95% CI, 0.27-1.23),^65^ and significant small effect sizes were observed for attitudes (SMD, 0.21; 95% CI, 0.09-0.33; 2 studies; *I*^2^ = 0.0%)^72,88^ and knowledge (SMD, 0.25; 95% CI, 0.08-0.42; 4 studies, *I*^2^ = 0.0%),^65,70,72,116^ along with a very small effect size for behaviors (SMD, 0.13; 95% CI, 0.03-0.22; 5 studies; *I*^2^ = 0.0%).^65,70,72,88,116^

Moderation analyses for short-term effects of active vs control interventions for attitudes, knowledge, and general stigma found no significant subgroup differences (eTables 3-5 in Supplement 1). In contrast, significant subgroup differences were found for behaviors (eTable 6 in Supplement 1): social contact interventions had larger effect sizes than educational approaches (β = 0.15; 95% CI, 0.00-0.30; *P* = .045).

Short-term comparisons between active groups showed significant small effect sizes for knowledge (SMD, 0.33; 95% CI, 0.16-0.50; 9 studies; *I*^2^ = 0.0%)^70,73,101,102,103,104,105,106,107^ and attitudes (SMD, 0.30; 95% CI, 0.02-0.57; 12 studies; *I*^2^ = 30.9%)^55,56,57,58,59,60,73,84,96,105,106,107^ (Figure 2).

Moderation analyses for short-term effects between active groups on attitudes found no significant subgroup differences (eTable 7 in Supplement 1). However, significant subgroup differences were found for behaviors (eTable 8 in Supplement 1): mixed-format interventions (ie, video and nonvideo based) had smaller effect sizes than video-based formats alone (β = −0.43; 95% CI, −0.83 to −0.04; *P* = .03), while nonvideo-based formats had larger effect sizes than video-based formats alone (β = 1.54; 95% CI, 0.04-2.67; *P* = .008). Forest plots of meta-analyses of stigma-related outcomes are presented in eFigures 1 to 6 in Supplement 1.

#### Outcomes Associated With Help-Seeking

As shown in Figure 2, meta-analyses of continuous data for short-term effects of active vs control interventions showed very small significant effect sizes for help-seeking attitudes (SMD, 0.18; 95% CI, 0.09-0.28; 9 studies; *I*^2^ = 0.0%)^67,69,75,95,97,108,112,114,124^ and intentions (SMD, 0.14; 95% CI, 0.07-0.21; 10 studies; *I*^2^ = 0.0%)^61,62,75,88,99,100,108,112,115,124^ and a significant long-term effect size for help-seeking attitudes (SMD, 0.15; 95% CI, 0.04-0.27; 2 studies; *I*^2^ = 0.0%).^88,114^ Two studies reporting dichotomous data showed significant short-term effects for help-seeking attitudes (OR, 1.77; 95% CI, 1.07-2.94; 2 studies; *I*^2^ = 0.0%)^108,120^ and midterm effects for formal help-seeking (OR, 1.99; 95% CI, 1.45-2.75; 2 studies; *I*^2^ = 7.9%)^63,111^ and informal help-seeking (OR, 2.24; 95% CI, 1.71-2.93; 1 study)^111^ (Figure 3). No significant effect sizes were found for short-term, midterm, or long-term effects of active vs active interventions.

Moderation analyses for short-term effects of intervention vs control conditions for help-seeking intentions found no significant subgroup differences (eTable 9 in Supplement 1). Forest plots of meta-analyses of outcomes associated with help-seeking are presented in eFigure 7 in Supplement 1.

#### Secondary Outcomes

No significant results were found for continuous secondary outcomes (eTable 10 in Supplement 1). A significant long-term benefit was observed between the intervention and control for helping behaviors using dichotomous data (OR, 1.43; 95% CI, 1.01-2.02; 3 studies; *I*^2^ = 0.0%)^65,109,114^ (eTable 11 in Supplement 1).

### Risk of Bias Across Studies

The funnel plots for short-term effects related to stigma are presented in eFigures 8 to 13 in Supplement 1. We found evidence of asymmetry in the distribution of effect sizes of active vs control interventions for attitudes, knowledge, and general stigma but not for behaviors. There was evidence of asymmetry in the distribution of effect sizes for comparisons between active groups on behaviors but not for attitudes. For help-seeking intentions, there was no evidence of asymmetry in the distribution of effect sizes for active vs control interventions (eFigure 14 in Supplement 1).

## Discussion

We systematically reviewed 97 studies to assess the outcomes associated with interventions designed to reduce mental health stigma among young people; 74 of these studies met the criteria for 3-level multivariate meta-analyses. These studies represent a diverse sample of 43 852 young people, predominantly from developed countries. Most studies focused on college students, with educational conferences and social contact interventions being the most common approaches. Our main findings indicate that interventions yielded small to medium significant improvements in stigma-related knowledge, attitudes, behaviors, and general stigma and very small but significant improvements in help-seeking attitudes and intentions, particularly in the short term. However, the improvements tended to diminish over time, highlighting the need for sustained efforts to maintain these gains.

Our findings align with previous systematic reviews supporting the immediate benefits of mental health stigma interventions on stigma-related outcomes among young people.^5,9,11,13,126^ The noticeable decline in positive associations over time observed in these outcomes highlights the challenges in maintaining the benefits of stigma reduction interventions, suggesting that short-term interventions may not be sufficient to produce lasting changes and that ongoing antistigma efforts, booster sessions, or follow-up activities may be necessary to sustain and enhance the initial gains.^6^ Interestingly, social contact and nonvideo-based interventions were found to have larger effect sizes for stigma-related behaviors than educational and video-based approaches, respectively. These findings reinforce the value of incorporating direct, personal interactions with individuals with mental illness into antistigma programs compared with less personal methods.^10,127,128,129^

Our meta-analyses of a small subset of studies indicate that stigma reduction efforts may positively influence young people’s willingness to seek help for mental health issues with the potential to translate into behavioral shifts.^130^ However, effect sizes were very small, with limited evidence for long-term improvements. The limited impacts may be attributed to persistent structural barriers and cultural factors that deter readiness and ability to seek mental health support, including fears of stigma and concerns about confidentiality.^21^ These findings highlight an important gap in antistigma interventions for promoting help-seeking behaviors. Importantly, as informal sources of help may be key in helping young people access mental health care,^131^ more studies should consider informal help-seeking as an outcome. Complementarily, the limited significant findings related to secondary outcomes may reflect the narrower focus of current intervention designs and stress the need for more comprehensive approaches that foster long-term resilience and recovery.^132^

An important strength of our systematic review was the use of 3-level multivariate meta-analyses. This method provides a more nuanced understanding of within- and between-study variability; improves the precision of estimates relative to univariable meta-analyses particularly for effect sizes with higher within-study heterogeneity; and uses all available information in the primary studies, a practice often neglected in univariable meta-analyses in which researchers may be forced to opt for a particular outcome measure.^18,19^

The findings of this review have several implications. First, the efficacy of social contact interventions suggests that integrating lived experience narratives may be a powerful tool for reducing stigma among young people. Second, the limited long-term effects highlight the necessity of sustained and ongoing antistigma efforts that reinforce initial gains. Third, the need for more robust measures of help-seeking behaviors (ie, formal, informal) and broader psychosocial outcomes (eg, empowerment, self-efficacy) is evident. Fourth, future RCTs may benefit from using mixed-methods approaches to capture the complexity of stigma and help-seeking, providing further understanding of the factors influencing them.^133^ Fifth, antistigma interventions were scarce from developing countries and were not found in any articles published in Spanish. As stigma is a universal phenomenon with important variations in both time and place,^134^ more research is urgently needed in these countries where young people are particularly burdened.^135^ Sixth, the exploration of digital interventions, which were underrepresented in this review, may offer scalable and accessible solutions for young people worldwide, especially in challenging health contexts such as the COVID-19 pandemic. A recent study showed that COVID-19 may have had a positive influence on mental health stigma^136^; thus, further research on stigma and stigma interventions is needed in this new postpandemic era.

### Limitations

Important limitations of the evidence included in our review should be noted. The overall risk-of-bias assessment indicated frequent issues with masking of participants and personnel and outcome assessment, which could introduce performance and detection biases. These issues are common in behavioral intervention studies, where masking is inherently challenging.^137^ Additionally, the unclear risk of bias in allocation concealment in more than half of the included studies points to potential selection biases that may affect the validity of the findings. Despite these limitations, the high percentage of studies with low risk of bias in random sequence generation and selective outcome reporting enhances the credibility of the reported effects. Importantly, the presence of asymmetry in the distribution of effect sizes suggests that studies with nonsignificant or negative results may be underreported, potentially inflating the observed effectiveness of interventions.

The reviewing process also had some limitations. Inclusion was limited to studies published in English or Spanish, potentially excluding important research presented in other languages. Grey literature was not included, which might have contributed to publication bias.

## Conclusions

In this systematic review and meta-analysis of 97 RCTs, we found that mental health stigma interventions for youth were associated with short-term reductions in stigma-related outcomes and improvements in help-seeking attitudes and intentions. This study provides a comprehensive overview of the current landscape of interventions aimed at reducing mental health stigma and enhancing help-seeking behaviors among young people. While the findings are encouraging, they also underscore the need for more rigorous long-term studies and the integration of multifaceted intervention strategies. By addressing these gaps, future research may contribute to the development of more effective, sustainable solutions to combat mental health stigma and support the well-being of young people globally.

## Notes

Supplement 1. **eAppendix 1.** Protocol Amendments **eAppendix 2.** Search Strategy **eTable 1.** Characteristics of the Studies **eTable 2.** Risk of Bias Within Studies **eTable 3.** Univariable Multilevel Meta-Regression Analyses of Selected Study Characteristics for Short-Term Effects of Interventions vs Controls on Stigma-Related Attitudes **eTable 4.** Univariable Multilevel Meta-Regression Analyses of Selected Study Characteristics for Short-Term Effects of Interventions vs Controls on Stigma-Related Knowledge **eTable 5.** Univariable Multilevel Meta-Regression Analyses of Selected Study Characteristics for Short-Term Effects of Interventions vs Controls on General Stigma **eTable 6.** Univariable Multilevel Meta-Regression Analyses of Selected Study Characteristics for Short-Term Effects of Interventions vs Controls Stigma-Related Behaviors **eTable 7.** Univariable Multilevel Meta-Regression Analyses of Selected Study Characteristics for Short-Term Effects Between Active Interventions Stigma-Related Attitudes **eTable 8.** Univariable Multilevel Meta-Regression Analyses of Selected Study Characteristics for Short-Term Effects Between Active Interventions Stigma-Related Behaviors **eTable 9.** Univariable Multilevel Meta-Regression Analyses of Selected Study Characteristics for Short-Term Effects of Interventions vs Controls on Help-Seeking Intentions **eTable 10.** Meta-Analytic Estimates for Secondary Outcomes (Continuous Data) **eTable 11.** Meta-Analytic Estimates for Secondary Outcomes (Dichotomous Data) **eFigure 1.** Forest Plot of Short-Term Effects of Interventions vs Controls on Stigma-Related Attitudes **eFigure 2.** Forest Plot of Short-Term Effects of Interventions vs Controls on Stigma-Related Behaviors **eFigure 3.** Forest Plot of Short-Term Effects of Interventions vs Controls on Stigma-Related Knowledge **eFigure 4.** Forest Plot of Short-Term Effects of Interventions vs Controls on General Stigma **eFigure 5.** Forest Plot of Short-Term Effects Between Active Interventions on Stigma-Related Attitudes **eFigure 6.** Forest Plot of Short-Term Effects Between Active Interventions on Stigma-Related Behaviors **eFigure 7.** Forest Plot of Short-Term Effects of Interventions vs Controls on Help-Seeking Intentions **eFigure 8.** Funnel Plot of Short-Term Effects of Interventions vs Controls on Stigma-Related Attitudes **eFigure 9.** Funnel Plot of Short-Term Effects of Interventions vs Controls on Stigma-Related Behaviors **eFigure 10.** Funnel Plot of Short-Term Effects of Interventions vs Controls on Stigma-Related Knowledge **eFigure 11.** Funnel Plot of Short-Term Effects of Interventions vs Controls on General Stigma **eFigure 12.** Funnel Plot of Short-Term Effects Between Active Interventions on Stigma-Related Attitudes **eFigure 13.** Funnel Plot of Short-Term Effects Between Active Interventions on Stigma-Related Behaviors **eFigure 14.** Funnel Plot of Short-Term Effects of Interventions vs Controls on Health-Seeking Intentions

Supplement 2.
Data Sharing Statement
