# Supplementary material for: Interventions to Reduce Mental Health Stigma in Young People: A Systematic Review and Meta-Analysis
Source: JAMA Netw Open. 2025 Jan 15;8(1):e2454730. doi: 10.1001/jamanetworkopen.2024.54730 (PMC11736514; doi:10.1001/jamanetworkopen.2024.54730)
Supplement: Supplement 1. — eAppendix 1. Protocol Amendments eAppendix 2. Search Strategy eTable 1. Characteristics of the Studies eTable 2. Risk of Bias Within Studies eTable 3. Univariable Multilevel Meta-Regression Analyses of Selected Study Characteristics for Short-Term Effects of Interventions vs Controls on Stigma-Related Attitudes eTable 4. Univariable Multilevel Meta-Regression Analyses of Selected Study Characteristics for Short-Term Effects of Interventions vs Controls on Stigma-Related Knowledge eTable 5. Univariable Multilevel Meta-Regression Analyses of Selected Study Characteristics for Short-Term Effects of Interventions vs Controls on General Stigma eTable 6. Univariable Multilevel Meta-Regression Analyses of Selected Study Characteristics for Short-Term Effects of Interventions vs Controls Stigma-Related Behaviors eTable 7. Univariable Multilevel Meta-Regression Analyses of Selected Study Characteristics for Short-Term Effects Between Active Interventions Stigma-Related Attitudes eTable 8. Univariable Multilevel Meta-Regression Analyses of Selected Study Characteristics for Short-Term Effects Between Active Interventions Stigma-Related Behaviors eTable 9. Univariable Multilevel Meta-Regression Analyses of Selected Study Characteristics for Short-Term Effects of Interventions vs Controls on Help-Seeking Intentions eTable 10. Meta-Analytic Estimates for Secondary Outcomes (Continuous Data) eTable 11. Meta-Analytic Estimates for Secondary Outcomes (Dichotomous Data) eFigure 1. Forest Plot of Short-Term Effects of Interventions vs Controls on Stigma-Related Attitudes eFigure 2. Forest Plot of Short-Term Effects of Interventions vs Controls on Stigma-Related Behaviors eFigure 3. Forest Plot of Short-Term Effects of Interventions vs Controls on Stigma-Related Knowledge eFigure 4. Forest Plot of Short-Term Effects of Interventions vs Controls on General Stigma eFigure 5. Forest Plot of Short-Term Effects Between Active Interventions on Stigma-Related Attitudes eFigure 6. Forest Plot [file jamanetwopen-e2454730-s001.pdf]

Supplemental Online Content

Crockett MA, Núñez D, Martínez P, et al. Interventions to reduce mental health stigma in young people: a systematic review and meta-analysis. *JAMA Netw Open*. 2025;8(1):e2454730. doi:10.1001/jamanetworkopen.2024.54730

eAppendix 1. Protocol Amendments

eAppendix 2. Search Strategy

eTable 1. Characteristics of the Studies

eTable 2. Risk of Bias Within Studies

eTable 3. Univariable Multilevel Meta-Regression Analyses of Selected Study Characteristics for Short-Term Effects of Interventions vs Controls on Stigma-Related Attitudes

eTable 4. Univariable Multilevel Meta-Regression Analyses of Selected Study Characteristics for Short-Term Effects of Interventions vs Controls on Stigma-Related Knowledge

eTable 5. Univariable Multilevel Meta-Regression Analyses of Selected Study Characteristics for Short-Term Effects of Interventions vs Controls on General Stigma

eTable 6. Univariable Multilevel Meta-Regression Analyses of Selected Study Characteristics for Short-Term Effects of Interventions vs Controls Stigma-Related Behaviors

eTable 7. Univariable Multilevel Meta-Regression Analyses of Selected Study Characteristics for Short-Term Effects Between Active Interventions Stigma-Related Attitudes

eTable 8. Univariable Multilevel Meta-Regression Analyses of Selected Study Characteristics for Short-Term Effects Between Active Interventions Stigma-Related Behaviors

eTable 9. Univariable Multilevel Meta-Regression Analyses of Selected Study Characteristics for Short-Term Effects of Interventions vs Controls on Help-Seeking Intentions

eTable 10. Meta-Analytic Estimates for Secondary Outcomes (Continuous Data)

eTable 11. Meta-Analytic Estimates for Secondary Outcomes (Dichotomous Data)

eFigure 1. Forest Plot of Short-Term Effects of Interventions vs Controls on Stigma-Related Attitudes

eFigure 2. Forest Plot of Short-Term Effects of Interventions vs Controls on Stigma-Related Behaviors

eFigure 3. Forest Plot of Short-Term Effects of Interventions vs Controls on Stigma-Related Knowledge

© 2025 Crockett MA et al. *JAMA Network Open.*

eFigure 4. Forest Plot of Short-Term Effects of Interventions vs Controls on General Stigma

eFigure 5. Forest Plot of Short-Term Effects Between Active Interventions on Stigma-Related Attitudes

eFigure 6. Forest Plot of Short-Term Effects Between Active Interventions on Stigma-Related Behaviors

eFigure 7. Forest Plot of Short-Term Effects of Interventions vs Controls on Help-Seeking Intentions

eFigure 8. Funnel Plot of Short-Term Effects of Interventions vs Controls on Stigma-Related Attitudes

eFigure 9. Funnel Plot of Short-Term Effects of Interventions vs Controls on Stigma-Related Behaviors

eFigure 10. Funnel Plot of Short-Term Effects of Interventions vs Controls on Stigma-Related Knowledge

eFigure 11. Funnel Plot of Short-Term Effects of Interventions vs Controls on General Stigma

eFigure 12. Funnel Plot of Short-Term Effects Between Active Interventions on Stigma-Related Attitudes

eFigure 13. Funnel Plot of Short-Term Effects Between Active Interventions on Stigma-Related Behaviors

eFigure 14. Funnel Plot of Short-Term Effects of Interventions vs Controls on Health-Seeking Intentions

This supplemental material has been provided by the authors to give readers additional information about their work.

© 2025 Crockett MA et al. *JAMA Network Open.*

eAppendix 1. Protocol Amendments

The following amendments were made to the protocol of this systematic review and meta-analysis PROSPERO registration number CRD42020210901.

Section	Old text	New text	Rationale for change
Participants/ population	Adolescents and young-adults –defined as people between 10 and 24 years of age–, their parents and/or caregivers, and educational/health professionals.	Adolescents and young adults, defined as people between 10 and 24 years of age.	Few studies had outcomes reported by parents, caregivers, educational, and/or health professionals.
Participants/ population	However, studies that include individuals with mental disorders will only be considered if these participants are identified as such through standardized procedures (e.g., application of validated self-report questionnaires), diagnostic evaluation by a professional psychologist or psychiatrist (e.g., clinical interview or semi-structured interview for these purposes), and/or review of clinical records.	–	Few studies used samples with mental disorders. Those who did include clinical samples not necessarily assessed or reported the quality of the diagnosis instrument/procedure.
Main outcome(s)	Primary outcomes will include information from post-intervention and/or follow-up evaluations of knowledge, attitudes, and/or behaviors (i.e. stereotypes, prejudices, and/or discriminatory behaviors) towards mental disorders, as well as help-seeking behaviors or access to mental health services	Based on a social-cognitive model of stigma and a framework for help-seeking for mental health problems (Rickwood & Thomas, 2012; Sheehan et al 2017). Primary outcomes were defined as stigma-related knowledge, attitudes, behaviors, and a general factor; and, help-seeking attitudes, intentions, and behaviors, further divided into formal (e.g., health professionals) and informal (e.g., friends) sources.	Because of the heterogeneity of outcomes related to stigma and help-seeking, conceptual models were used to more clearly determine the type of outcome assessed.
Main outcome(s)	Outcomes must have been assessed with validated instruments	–	This information is not frequently reported by the studies.
Additional outcomes(s)	Outcomes must have been assessed with validated instruments	–	This information is not frequently reported by the studies.
Strategy for data synthesis	If the performance of a meta-analysis is feasible, then,	If the performance of a meta-analysis is	The analysis was updated to consider as much information

© 2025 Crockett MA et al. *JAMA Network Open.*

	considering recent developments on this technique, a meta-analysis of heterogeneous studies will be conducted using the quality effects estimator, which gives greater weight to large and better-quality studies (Doi et al., 2015).	feasible, then, a three-level multivariate meta-analysis will be conducted to account for within-study correlations and maximize data utilization (Riley, 2009; Wei, 2013).	as possible from the articles, considering the possible relationship of outcomes within each study.
Strategy for data synthesis	Statistical analyses will be performed with MetaXL, a free Excel plugin for meta-analysis (Barendregt & Doi, 2016).	Statistical analyses will be performed with the "metafor" package in R 4.2.0 (Viechtbauer, 2010).	The statistical software was updated to perform the three-level multivariate meta-analysis.

© 2025 Crockett MA et al. *JAMA Network Open.*

eAppendix 2. Search Strategy

1. CENTRAL
(stigma:ti,ab OR [mh "social stigma"] OR [mh "prejudice"] OR [mh "stereotyping"] OR [mh "social discrimination"]) AND (mental disorders:ti,ab OR mental health:ti,ab OR mental illness:ti,ab OR psychopathology:ti,ab OR [mh "mental disorders"] OR [mh "mental health"] OR [mh "psychopathology"]) AND (child:ti,ab OR adolescent:ti,ab OR youth:ti,ab OR [mh "child"] OR [mh "adolescent"] OR [mh "young adult"])

2. CINAHL
((TI Stigma) OR (AB Stigma) OR (MH "Stigma") OR (MH "Prejudice") OR (MH "Stereotyping") OR (MH "Discrimination+")) AND ((TI Mental Disorders) OR (AB Mental Disorders) OR (TI Mental Health) OR (AB Mental Health) OR (TI Mental Illness) OR (AB Mental Illness) OR (TI Psychopathology) OR (AB Psychopathology) OR (MH "Mental Disorders+") OR (MH "Mental Health+") OR (MH "Psychopathology+")) AND ((TI Child) OR (AB Child) OR (TI Adolescent) OR (AB Adolescent) OR (TI Youth) OR (AB Youth) OR (MH "Child+") OR (MH "Adolescence+") OR (MH "Young Adult+")) AND ((MH "clinical trials+") OR (MH "interrupted time series analysis") OR (MH "controlled before-after studies") OR (MH "quasi-experimental studies+"))

3. EMBASE
("stigma":ab,ti OR "stigma"/exp OR "social stigma"/exp OR "prejudice"/exp OR "stereotyping"/exp OR "social discrimination"/exp) AND ("mental disorders":ab,ti OR "mental health":ab,ti OR "mental illness":ab,ti OR "psychopathology":ab,ti OR "mental disease"/exp OR "mental health"/exp) AND ("child":ab,ti OR "adolescent":ab,ti OR "youth":ab,ti OR "child"/exp OR "adolescent"/exp OR "young adult"/exp) AND ("clinical trial"/exp OR "clinical trial (topic)"/exp OR "interrupted time series":ab,ti OR "controlled before-after stud*":ab,ti OR "quasi experimental study"/exp)

4. PubMed
(Stigma[Title/Abstract] OR "Social Stigma"[Mesh] OR Prejudice[Mesh] OR Stereotyping[Mesh] OR "Social Discrimination"[Mesh]) AND (Mental Disorders[Title/Abstract] OR Mental Health[Title/Abstract] OR Mental Illness[Title/Abstract] OR Psychopathology[Title/Abstract] OR "Mental Disorders"[Mesh] OR "Mental Health"[Mesh] OR "Psychopathology"[Mesh]) AND (Child[Title/Abstract] OR Adolescent[Title/Abstract] OR Youth[Title/Abstract] OR Child[Mesh] OR Adolescent[Mesh] OR "Young Adult"[Mesh]) AND ("Clinical Trial"[Publication Type] OR "Clinical Trials as Topic"[Mesh] OR "Interrupted Time Series Analysis"[Mesh] OR "Controlled Before-After Studies"[Mesh] OR Quasi-experimental[Title/Abstract])

© 2025 Crockett MA et al. *JAMA Network Open.*

eTable 1. Characteristics of the Studies

Study ID	Study design	Participant	Intervention	Comparator	Outcomes
Amsalem, 2022a (US)*[98]	RCT - Individual	1098 young participants from a crowdsourcing platform, mean age = 16.9 (SD = 1.2, range = 14-18), 43% female, 44% male, and 13% non-binary, trans and prefer not to say.	Brief videos (114-118 seconds) of transgender girl (n = 393) and transgender boy (n = 391) about their personal story about coping with depression and seeking help.	Two brief control videos (102-113 seconds) with a cisgender female, n = 166, and male protagonists, n = 148, about how they coped with depression and recovered with help and support.	Stigma towards depression (DSS), measured at baseline and post-intervention.
Amsalem, 2022b (US)*[100]	RCT - Individual	1183 young participants from a crowdsourcing platform, mean age = 16.8 (SD = 1.2, range = 14-18), 48% female, and 52% male.	Brief videos (102-113 seconds) of a girl (n = 386) and a boy (n = 395) about coping with depression and seeking help.	Two control videos (100 seconds) with a girl and a boy about their hobbies, n = 208.	Stigma towards depression (DSS), and treatment-seeking intentions (GHSQ), measured at baseline and post-intervention.
Amsalem, 2023a (US)*[99]	RCT - Individual	Study 1: 895 young adults from a crowdsourcing platform, mean age = 23.9 (SD = 3.7, range = 18-30), 48% female, and 52% male. Study 2: 637 adolescents from a crowdsourcing platform, mean age = 17.1 (SD = 1.1, range = 14-18), 49% female, and 51% male.	Study 1: Two brief videos (75 seconds) about a young woman with schizophrenia who describe her disorder, struggles, and recovery, one in traditional format (n = 302) and other in a selfie format (n = 304). Study 2: two brief videos in different formats, traditional format (102 seconds, n = 220) and selfie video (58 seconds, n = 212), about a girl with depression and suicidal thoughts, and how treatment help her.	Study 1: non-intervention control, n = 289. Study 2: control video with the same girl talking about her family and interest, n = 205.	Study 1: public stigma toward people with schizophrenia, measured at baseline, post-intervention, and 30-day follow-up. Study 2: stigma toward depression (DSS) and treatment-seeking intentions (GHSQ), measured at baseline and post-intervention.
Amsalem, 2023c (US)[26]	RCT - Individual	1181 young adults from a crowdsourcin	Two brief videos (76 and 82 seconds) about a woman	Control group without	Social distance, stereotyping, separateness,

© 2025 Crockett MA et al. *JAMA Network Open.*

		g platform, mean age = 24.0 (SD = 3.6, range = 18-30), 48% female, and 52% male.	living with schizophrenia who described her struggles associated with her illness, her recovery and hope. One video was made from a gender perspective (n = 470) and the other is not (n = 469).	intervention, n = 242.	social restriction, and public perception of recovery, measured at baseline, post-intervention, and 30-day follow-up.
Boucher, 2014 (US)[27]	RCT - Individual	182 undergraduate students enrolled in introductory psychology courses, mean age = 20.5 years old, 63.7% women, 35.7% men, and 0.5% transgender.	Brief online message (billboards advertising) about depression as a brain disease, n = 91.	Brief online control message not related to health or mental health, n = 91	Stigma (AQ and SSRPH), attitudes (EQ), and help-seeking, measured at baseline and post-intervention.
Brown, 2010a (US)*[56]	RCT - Individual	127 undergraduate students, mean age = 18.7 years old (SD = 0.9), 50.4% female, and 49.6% male.	Brief simulations (16 min) of auditory hallucinations sitting in a research lab, n = 65.	Brief simulations of auditory hallucinations ambulating around a college campus, 16 min, n = 62	Stigma (AQ) measured at baseline and post-intervention.
Brown, 2010b (US)*[84]	RCT - Individual	143 undergraduate students, mean age = 19.3 years old, 58% female, and 42% male.	Group 1: A 16-minutes film with three psychiatrically stable individuals (diagnosed with schizophrenia) discuss their symptoms, difficulties, treatment, and accomplishments, n = 40; Group 2: A 16-minutes audio segment to simulate a credible experience of auditory hallucinations, n = 58.	Control group without intervention, n = 45	Social distance (SDS), negative emotions towards people with mental illness (AS), measured at baseline, post-intervention, and 1 week follow-up.

© 2025 Crockett MA et al. *JAMA Network Open.*

Brown, 2017 (US)*[71]	RCT - Individual	424 undergraduate students enrolled in introductory psychology courses, mean age = 19 years old, 48.6% female, 51.4% male, and 20% reported previous mental health treatment.	Viewing of a 90-seconds ad for medication prescribed for bipolar disorder (Latuda; 90 seconds long); information about the disorder and side effects, n = 214.	Viewing of a 90-seconds automobile ad, n = 210.	Beliefs about bipolar disorder (made for study) and stigma (SDS, DS, AS, FTS), measure baseline and post-intervention.
Bruins, 2018 (NL)*[91]	RCT - Cluster (classes)	89 high school students from 5 classes, mean age = 16.3 years old (SD = 0.49), 47.2% female, and 52.8% male.	Intervention, 45 mins, delivered once experimental documentary focused on normalizing psychotic experiences and it featured a 16-year-old male adolescent with schizophrenia, who spends the day with a female Dutch celebrity (‘Are you Crazy?!’ In Dutch: ‘Ben jij gek?!’), n = 58.	Control scene from ‘March of the Penguins’ (45 min) delivered once, n = 31.	Stigmatizing attitudes (MISS) measured at baseline and post-intervention.
Campbell, 2011 (UK)[28]	RCT - Cluster (classes)	92 students from a secondary school, mean age = 14.6 years old, 68.5% female, and 31.5% male.	Workshop to normalize psychosis with interactive/informative presentation on mental health, and a service user researcher presenting his psychotic-like experiences, n = 43.	Control group without intervention, n = 49.	Discrimination (AQ), measured at baseline, post-intervention, and 10 weeks follow-up.
Campos, 2018 (PT)*[64]	RCT - Cluster (classes)	543 students from third cycle of their basic education from 22 school classes, mean age = 13.04 years old (SD = 0.79, range	Finding Space, two 90-min sessions delivered at one-week. The intervention followed an interactive methodology using group dynamics, music, and videos, 11 classes, n = 259.	Control delayed intervention, 11 classes, n = 284.	Mental health literacy (MHLq) measured at baseline, post-intervention, and 6-months follow-up.

© 2025 Crockett MA et al. *JAMA Network Open.*

		= 12-14), 48% female, and 52% male.			
Casañas, 2022 (ES)*[72]	RCT - Cluster	1032 students from 18 secondary schools, mean age = 14.2 (SD = 0.58, range = 13-16), 49.6% women, and 50.4% men..	EspaiJove.net consist in three educational programmes delivered by mental health nurses: Sensitivity Programme (1 hour, 3 schools, n = 225), Mental Health Literacy (6 hours, 5 schools, n = 261), and Mental Health Literacy plus a first-person Stigma Reduction Programme which included a social contact activity (7 hours, 5 schools, n = 295)	Waitlist, 5 schools, n = 251.	Mental health Knowledge (Espaijove Mental Health Literacy Test), behaviours (RIBS) and attitudes (CAMI) towards mental health stigma, help-seeking (GHSQ single item and instrument made for the study), measured at baseline, 2-weeks post-intervention, 6-months follow-up, and 12-months follow-up.
Chan, 2009 (CN)*[105]	RCT - Cluster (classes)	255 9th grade students from 3 secondary schools, mean age = 14.6 years old, 60% female, 40% male, and 1.2% reported having schizophrenia.	Two conditions Education-Video and Video-Education, changing sequence of components. Contact video (15 min.) featuring life experiences of four real persons diagnosed with schizophrenia, Education-Video n = 94. Video-Education n = 73.	Education to challenge myths about schizophrenia, providing comprehensive knowledge and factual information, 30 min, n = 88.	Stigma (PSS), social distance (SDS), and knowledge (KT), measured at baseline, post-intervention, and 1-month follow-up.
Cheetham, 2020 (AU)[29]	RCT - Cluster (schools)	463 9th grade students, mean age = 14.9 years old (range = 13-16), 76.5% female, and 23.5% male.	MAKINGtheLINK five interactive classroom activities, 7 schools, n = 196.	Control group without intervention, 7 schools, n = 165.	Stigma (Stigma scale) measured at baseline and three follow-ups that occurred 6-weeks, 6-months, and 12-months post-baseline.
Chisholm, 2016 (UK)*[101]	RCT - Cluster (classes)	657 8th year secondary school students from 31 classes, mean age =	Contact and education, single day, 6 hours with 20 mins of contact module, 16 classes, n = 405.	Education alone, single day, 6 hours, 15 classes, n = 364.	Intended behavior (RIBS), knowledge (MAKS), and help-seeking, measured

© 2025 Crockett MA et al. *JAMA Network Open.*

		12.2 years old (SD = 0.58; range = 11-13), 52.1% female, and 47.9% male.			baseline and 2 weeks follow-up.
Chung, 2004 (CN)[31]	RCT - Individual	313 secondary students, mean age = 15,2 years old (SD = 0,9), 49,5% female, 50.5% male, and 13,7% had previous contact with someone with a mental illness.	Students presented with a case vignette using the schizophrenia label (Schizophrenia: jing-shen-fen- lie-zheng) and students presented with a case using the Chinese label for psychosis (dysregulation of thought and perception: si-jue-shi-diao)	Students presented with a case using the mental illness label (Mental illness: jing-shen-bin) and students presented with a case using no label.	Social distance, stereotypes of schizophrenia, and attributions regarding mental illness (made for study), measured at the end of the intervention.
Chung, 2005 (CN)[30]	RCT - Cluster (rotations)	166 medical students in psychiatry clinical rotations, mean age = 22.8 years old, 39.8% female, and 60.2% male.	A 1-hour stigma change seminar consisting of informal contact with mental health consumers and education on misconceptions about psychiatric patients, three rotations, n = 81.	A clinical rotation in psychiatry without the seminar on psychiatric stigma, three rotations, n = 85.	Attitudes towards people with schizophrenia (ATP-30) measured at baseline and post-intervention.
Chung, 2022 (HK)*[73]	RCT - Individual	45 young adults recruited from tertiary educational institutions, mean age = 21.3 (SD = 0.65, range = 19-23), 68.9% female, 31.1% male.	The Human Library intervention (2 hours) were two persons who shared their stories, a woman with schizophrenia and a man with bipolar disorder, n = 15.	Teaching session about mental disorders (2 hours, n = 15) and a control group without intervention (n = 15).	Mental health knowledge, perceived stigma, and social distance (MHLq), measured at baseline and post-intervention.
Conceição, 2022 (PT)[32]	RCT - Individual	969 first-year college students, mean age = 19.9 (SD = 1.48), 60.7% woman, and 39.3% men.	A video with people sharing their personal experiences with depression (2 min and 56 s, n = 334), and the same video with a psychoeducational information video	Control group without intervention, n = 313.	Attitudes towards professional psychological help (ATSPPH) and stigma towards depression (DSS) measured at baseline, post-test, and 5-

© 2025 Crockett MA et al. *JAMA Network Open.*

			(2 min and 50 s, n = 322).		months follow-up.
Conceição, 2024 (PT)*[63]	RCT - Individual	702 first-year college students, mean age = 18.9 (SD = 1.87), 59.4% female, and 40.6% male.	A video with people sharing their personal experiences with depression (3 min) and a video with a psychoeducational information video (3 min, n = 235).	The first intervention video (3-min, n = 242); and a control group without intervention, n = 225	Access to mental health care (made for study), measured at baseline, post-intervention, 5-months, 13-months, and 22-months follow-up.
Corrigan, 2007 (US)*[106]	RCT - Individual	244 students and people of a Chicago area community college, mean age = 23.9 years old, 70.1% female, and 29.9% male.	Education videotape, where a person with mental illness reviewed and contrasted the myths of mental illness from the facts. The teacher did not disclose his mental illness, 10 minutes, n not reported.	Contact videotape about a person with schizophrenia about the experiences with symptoms and recovery, 10 minutes, n not reported.	Stigma related attitudes (AQ), assessed at baseline, post-intervention, and 1-week follow-up.
Corrigan, 2010 (US)[33]	RCT - Individual	200 participants of four colleges, mean age = 20.2 years old (SD = 2.96), 66,7% female, and 33.3% male.	Our Own Voice a 90 min group interaction (n = 66) or 30 min group interaction (n = 67). Both had five components related to mental illness experiences, distress, acceptance, treatment, coping strategies, and recovery.	Educational intervention in a 30 min single session, n = 67.	Perception and recollection of persons with severe mental illness (LSMT) measured post-intervention only.
Costin, 2009 (AU)*[108]	RCT - Individual	348 young adults drawn from the Australian community, age range = 19-24 years old, 70.6% female, and 22.4% male.	Basic intervention (depression information health e-cards). Enhanced depression information group (information about depression and help seeking, what to expect at consultation with each health professional, and tips about contacting health professionals and asking for help). Basic intervention low distress group n	Information about general health issues unrelated to depression, n low distress group = 65, n high distress group = 52.	Help-seeking behavior (AHSQ), help-seeking intentions (GHSQ), help-seeking knowledge, beliefs about help-seeking, and ability to recognize depression (made for study), measured at baseline and 6-weeks post-intervention.

© 2025 Crockett MA et al. *JAMA Network Open.*

			= 63, high distress group n = 51. Enhanced intervention low distress group n = 70, high distress group n = 47.		
Davies, 2018 (UK)*[94]	RCT - Individual	55 medical students, mean age = 19.9 years old (SD = 3.2), 65.5% female, 34.5% male, and 25% had personal experience of mental health problems.	MHFA eLearning, education about specific mental disorder and abilities to assist themselves and someone developing a mental health problem, 6 modules (6-8 hours) during 3-months, n = 27.	Control group without intervention, n = 28.	Mental health first aid intentions (made for study) and personal stigma (DSS), measured at baseline and 6 weeks follow-up.
Dzemaili, 2023 (CH)*[65]	RCT - Individual	107 second-year college students, mean age = 24.0 (SD = 4.17), 70.1% women, and 29.9% men.	MHFA, 12-hour face-to-face training program delivered in four sessions by two instructors. The activities included didactic lectures and role playing about how to recognize and react to signs and symptoms of mental health illness, n = 53.	Control group without intervention, n = 54.	Mental health knowledge (made for study), attitudes (DSS) and behaviors (SDS) towards mental illness, confidence to help, intention to help, and first aid actions (adapted for study), measured at baseline, 3-months, and 12-months follow-up.
Economou, 2012 (GR)[34]	RCT – Cluster (classes)	616 secondary school students recruited from 11 public schools, mean age = 13.8 years old (SD = 0.82), 51,4%girls, and 48.6% boys.	Two-hour semi structured face to face anti-stigma program delivered by two mental health professionals, n =308.	Talk about nutrition and healthy living, n = 308.	Beliefs, attitudes, and social distance (Alberta Pilot Site Questionnaire Toolkit), measured at baseline, post-intervention, and 12-month follow-up.
Economou, 2014 (GR)[35]	RCT – Cluster (classes)	1081 students form 20 public schools, age	Anti-stigma intervention about severe mental illness using	2-hour discussion about	Beliefs, attitudes, and social distance (Alberta Pilot Site

		range = 13-15 years old (89% of sample was 14 years old), 53.1% female, and 46.9% male.	psychoeducation and case vignettes, 2 hours, n = 551.	immunization, n = 530.	Questionnaire Toolkit), measured at baseline and post-intervention.
Esanu, 2016 (PT)[36]	RCT – Cluster (schools)	240 10th grade students from 8 schools, mean age = 15.8 years old.	A two-hour single session of educational intervention, n not reported.	Control group without intervention, n not reported	Stigma (AQ) measured at baseline, post-intervention, and 6 months follow-up.
Farina, 2020 (UK)*[74]	RCT - Individual	319 secondary students from three schools, mean age = 12.6 years old (range = 12-16), 57.1% female, and 42.9% male.	Dementia Friends, psychoeducational intervention covering five key messages that everyone should know about dementia, 45-60 minutes per session, n = 198.	Curriculum as usual, n = 102.	Attitudes (Brief A-ADS) and understanding of dementia (KIDS), and willingness to work with people with dementia (made for study), measured at baseline and post-intervention.
Fernandez, 2016 (MY)*[57]	RCT - Individual	102 pre-clinical medical students, age range = 20-23 years old, 78% female, and 22% male.	A 45-min face-to-face contact (personal testimony on a patient´s recovery story, experience of stigma, experiences with mental illness, and his encounters with the mental healthcare system in Malaysia) and 90-min educational lecture including an overview of mental illness, n = 51.	40-min video-based contact and 90-min educational lecture, n = 51.	Stigma (OMS-HC), measured at baseline, post-intervention, and 1-month follow-up.
Finkelstein, 2008 (RU)*[55]	RCT - Individual	193 graduate students, mean age = 19.1 years old (SD = 1.7), 99% female, and 1% male.	Anti-stigma computer program providing short educational messages addressing cognitive, emotional, and behavioral components of stigma towards severe mental disorders, n = 69.	Reading group (n = 76) with unbiased and introductory facts on psychiatric disorders and stigma, having the same data as the computer program. Control condition without	Social distance (BSSD), stigmatizing attitudes (CAMI), and knowledge (PKS), measured at baseline, post-intervention, and 6 months follow-up.

				intervention (n = 48).	
Giannakopoulos, 2012 (GR)*[85]	RCT - Cluster (schools)	161 students from two public high schools in a municipality in Athens, mean age = 16.8 years old, 51.2% female, and 48.8% male.	Three weekly 90-minute workshops provided by two trained mental health professionals about educating on mental health, challenging stigma, and enhancing attitudes toward the mentally ill, n = 86.	Control condition without intervention, n = 75.	Stigmatizing attitudes (CAMI) and social distance (FABI), measured at baseline and 1-month follow-up.
Grandón, 2023 (CL)*[86]	RCT - Individual	244 college students from medicine, nursing, dentistry, obstetrics, psychology, and social work, mean age = 20.9 (SD = 1.97), 74.2% female, and 25.8% male.	Adaptation of Equal-Mind programme, that included education and social contact activities in four sessions, n = 123.	Control group without intervention, n = 121.	Mental health stigma (EAPS-TM and SDS), measured at baseline and 10-days post-intervention.
Greif Green, 2020 (US)*[51]	RCT - Individual	52 students in undergraduate and graduate preservice teacher programs, 50% were between 18-20 years old, 30.3% were between 21-25 years old.	Kognito Training: teachers learn specific strategies for discussing mental health with students and conduct mental health referrals. Delivered virtually to educators. n = 28.	Training from the IRIS Center, providing online resources to support the use of effective practices and interventions for students, n = 24	Gatekeeping behavior (Gatekeeping Behavior Scale), Intended behavior (RIBS), measured twice during session 1 (pre- and post-survey) and 1-month follow-up.
Gulliver, 2012 (AU)*[75]	RCT - Individual	120 elite athletes from the Australian Institute of Sport and from other national sporting organizations in Australia, mean age 25.4 (SD = 5.6), 73% female, and 27% male.	Written material delivered on 34 brief linear webpages, to increase mental health literacy and decrease stigma on depression and anxiety (n = 31); Two interactive quizzes providing tailored feedback to the participant about their level of depression and anxiety (n = 30);	Control condition without intervention, n = 29.	Help-seeking attitudes (ATSPPHS-SF), help-seeking intentions (GHSQ), mental health literacy (D-Lit, A-Lit), stigma toward depression (DSS), and stigma towards anxiety (GASS), measured at pre-intervention, post-intervention,

© 2025 Crockett MA et al. *JAMA Network Open.*

			Three pages each week, including an introduction, help-seeking source page, and a congratulatory page (n = 30).		and 3-month follow-up.
Han, 2006 (TW)*[61]	RCT - Individual	299 students in three universities, mean age = 20.3 years old, 73% female, and 27% male.	Three conditions. Biological Education (n = 75), students read information on the biological etiology of depression (5-10 min). Destigmatization (n = 76), students read information to reduce the psychological blameworthy towards depressed people (5-10 min). Combined (n = 72), biological education plus destigmatization.	Control condition without intervention, n = 76.	Willingness to seek help (HSWS), measured at baseline and 2-weeks follow-up.
Han, 2014 (TW)*[87]	RCT - Individual	132 undergraduate students enrolled in psychology courses, mean age = 19.6 years old, 63.6% female, and 36.4% male.	Computer-assisted psychoeducation on major depressive disorders, through illustrations of symptoms, courses, prognoses, treatments and help-seeking resources, 30 minutes, n = 66.	Control condition without intervention, n = 62.	Attitudes toward biological attributions of depression (BAS), psychological blame (PBS), social distance (DSD) measured at baseline and 2-weeks follow-up.
Han, 2018 (TW)[37]	RCT - Individual	257 Australian and Chinese university students, age range = 18-30 years old, 61.9% female, and 38.1% male.	Online psychoeducational program that consists in two modules assessing suicide literacy (module 1) and self-reliance, social support, and myths about mental health professionals (module 2), 5 min per module, n = 125.	Two 5-min modules about healthy lifestyles and no content directly relevant to suicide, n = 132.	Professional help-seeking beliefs and intentions (GHSQ), professional help-seeking attitudes (ATSPPHS-SF), suicide stigma (Stigma of Suicide Scale short-form), measured at baseline, post-intervention, and 1-month follow-up.

Han, 2023 (CN)*[102]	RCT - Individual	144 young LGBTQ+ adults with moderate or above symptoms of depression, anxiety and/or stress, mean age = 22.2 (SD = 2.8), 27.5% cisgender female, 23.2% cisgender male, 49.3% transgender, non-binary, and other.	Online intervention composed of psychoeducational videos, online facilitator-led group discussion, and electronic brochures, n = 72.	Control group with same online and length intervention with no mental help-seeking contents, n = 72.	Help-seeking intentions (GHSQ), attitudes toward seeking professional help (ATSPPH-SF), actual help-seeking behavior (AHSQ), depression and anxiety literacy (D-Lit and A-Lit), self-stigma toward seeking professional help (SSOH), and help-seeking encouragement (HSERK), measured at baseline, post-intervention, 1-month, and 3-months follow-up.
Hart, 2018 and 2022 (AU)*[88,109]	RCT - Cluster	1942 year 10 students, mean age = 15.9 (SD = 0.5), 44.7% female, and 55.3% male.	tMHFA, delivered in three 75-min sessions, n = 989.	PFA, delivered in three 75-minure sessions, n = 953.	Quality of MHFA intentions, confidence to help, problem recognition, quality of first aid behaviors, beliefs about helpfulness of adult help, help-seeking intentions (made for study), and stigmatising beliefs (SDS, DSS), measured at baseline, post-intervention and 12-months follow-up.
Howard, 2018 (AU)*[110]	RCT - Individual	350 senior high school students, median age = 16 years old, 47% female, 53% male, and 39% had clinically significant depressive symptoms	One time during school class time (40 min) delivered online information about criteria for major depression with educational information on biological causes of depression on a website, n = 135.	One 40-min class about criteria and psychosocial causes for major depression (n = 49, only female); one 40-min class about criteria and symptoms	Self-stigma (SSDS), social distance (SDS), help-seeking intentions (GHSQ), measured at baseline and post-intervention.

		(score 16+ in CES-D).		for major depression (n = 119).	
Jorm, 2010 (AU)*[111]	RCT - Cluster (schools)	423 8th to 10th grade students from 16 schools, 327 teachers in 14 schools were included, students age range = 12-15 years old (38% were 13 years old), 54% female students, 46% male students, 65.1% female teachers, and 34.9% male teachers.	MHFA, two one-day parts of seven hours each, 7 schools, n = 221.	Waitlist, 7 schools, n = 106.	Knowledge about mental health problems, beliefs about treatment of depression, recognition of depression and stigma towards depression (teachers). Recognition of depression, help-seeking intentions and beliefs, stigma, help received from teacher (students). Measured at baseline, post-intervention, and 6-months follow-up.
Kerby, 2008 (UK)*[89]	RCT - Individual	46 medical students, mean age = 21, 74% female, and 26% male.	Two films (15-min and 12-min) about people who had experienced mental illness, n = 23.	25-min control film unrelated to mental illness, n = 23.	General attitudes to serious mental illness (ATSMI-AV), perceived dangerousness (DS), social distance (SDS), attitudes to psychiatry (APS), and behavioral intentions towards people diagnosed with a mental illness (made for study), measured at baseline, post-intervention and 8-weeks follow-up.
King, 2015 (US)*[58]	RCT - Individual	76 college students, mean age = 22.9 years old (SD = 5.0),59.1% women, 40.9% men.	eBridge received personalized feedback based on motivational interview principles after baseline measures, and students could connect with an	Control condition received feedback after baseline measures but did not have the option to contact with the study team	Professional help-seeking, public stigma (DSS item), personal stigma (DSS item), and readiness to access help, measured at baseline and 2-

© 2025 Crockett MA et al. *JAMA Network Open.*

			online counselor, n = 41.	(online counseling or otherwise) related to their feedback or mental health resources, n = 35.	months follow-up.
Kirschner, 2022 (US)*[95]	RCT - Individual	85 college students, mean age = 19 (SD = 1.5, range = 18-25), 76.7% female, and 23.3% male.	30-minutes online interactive educational intervention that utilizes avatars depicting distressed individuals designed to help college students to identify, approach, and refer at-risk peer for help, n = 30.	30-min online interactive educational program about tobacco use and sales among minors, n = 30; and a assessment control condition, n = 25, that only answer post-intervention.	Attitudes toward seeking professional psychological help (ATSPPH), self-stigma of seeking help (SSOSH), and Perceptions of stigmatization by others for seeking help (PSOSH), measured at baseline and post-intervention.
Koike, 2016 and Yamaguchi, 2019 (JP)*[38,70]	RCT - Individual	259 undergraduate and graduate students from 20 colleges and universities, mean age = 20 years old, 42.1% female, and 59.9% male.	Filmed social contact, 30-minute video (n = 89); Internet-based self-study, online single-session, 30 min (n = 83)	30-min video game session, n = 87.	Past and future intended behavior (RIBS-J) and knowledge of mental illness (MIDUS), measured at baseline, post-intervention, 1, 12, and, 24-months follow-up.
Kosyluk, 2016 (US)*[112]	RCT - Individual	198 psychology students, mean age = 21.3 years old, 36.9% female, 62.1% male, and 1.0% transgender and prefer not to say.	Group 1: Contact-based anti-stigma presentation (a student with a mental illness telling his or her story about mental illness), 15-20 minutes, n = 64. Group 2: Education-based presentation. 15-20 minutes, n = 66.	Ted Talk video about beatboxing, which discussed no issues related to mental illness or any other type of disability, 15-20 minutes, n = 68.	Social distance (SDS), personal stigma (AQ), perceived stigma (PDDS), label avoidance (SSOSH), help-seeking attitudes (ATSPPHS), and intentions (GHSQ), measured at baseline and post-intervention.
Kruger, 2022 (US)*[62]	RCT - Individual	163 undergraduate psychology students, mean age = 21.1 (SD =	25-min documentary designed for college students about same-age peers with	A 25-min clip of a documentary about a doctor living with schizophrenia	Intentions to seek counseling (ISCI), measured at baseline and post-intervention.

		2.2, range = 19-29), 74% female, and 26% male.	mental health concerns, n = 54.	and her recovery, n = 56; and a 25-min clip video unrelated to mental health, n = 53.	
Lannin, 2013 (US)[39]	RCT - Individual	84 undergraduate students at a midwestern university experiencing psychological distress (CORE-GP), mean age = 21.2 years old, 60% female, and 40% male.	Single session involving a 5-minutes self-affirmation writing task, designed to affirm positive core characteristics informing participants' self-worth, n not reported.	Individual single session involving a 5-minutes writing task in which participants described jellybean flavors, n not reported.	Self-stigma (SSOSH), intentions (ISCI) and willingness (WSHS) to seek psychotherapy, measured at baseline and post-intervention.
Lincoln, 2008 (DE)[40]	RCT - Individual	61 undergraduate psychology (mean age = 21.2 years old) and 60 medical students (mean age = 22.5 years old), 78.5% female, and 21.5% male.	Leaflets and videos of simulated patients highlighting a biogenetic etiology n = 41; psychosocial explanation of schizophrenia n = 40.	Information leaflet and video about glaciers, n = 39.	Implicit attitudes (IAT), explicit attitudes and social distance (made for study and SDS), measured at baseline and post-intervention.
Link, 2020 (US)[*113]	RCT – Cluster (classes)	484 6th grade students from 14 schools, mean age = 11.5 years old, 56% girls, 44% boys, and 18.5% of youth met criteria for a mental health symptoms checklist.	Curriculum module about stigma, mental health treatment, and specific mental disorders. 3 modules of 3 hours delivered within 1 week, 4 groups, n = 92.	Contact with two adults with psychiatric hospitalization experience, Printed material about people with mental illness. Contact only (3 groups, n = 85), Materials only (2 groups, n = 52). Curriculum and Contact (2 groups, n = 42), Curriculum and Materials (1 group, n = 24), Curriculum, Contact, and	Knowledge and attitudes (Knowledge and Positive attitudes), social distance (Children's social distance), mental health treatment seeking, measured at baseline, 3-weeks, 6-, 12-, 18-, and 24-months post-intervention.

				Materials (2 groups, n = 40), Contact and materials (2 groups, n = 40), No intervention (3 groups, n = 52).	
Lipson, 2014 (US)*[76]	RCT - Cluster (campus residences)	549 residential advisors (RAs) and 1965 residents (both undergraduate students) from 32 colleges and universities across the US, mean age = 19.4-20.4 years, 57.5-63.9% women, and 36.1-42.5% men.	Gatekeeper (RAs) training in a 12-hour mental health first aid program to encourage self-management and help-seeking, plus pre-existing mental health training. RAs = 291, residents = 988.	Pre-existing mental health training at the institution, RAs = 262, residents = 1002.	Knowledge (single item), stigma (D-DS), and help-seeking, measured at baseline and 2-3 months follow-up.
Lubman, 2020 (AU)*[114]	RCT - Cluster (schools)	2447 students from 22 secondary government, catholic, and independent schools, mean age = 14.9 years old, 50% female, and 50% male, 47.6% previously sought help for any mental health problem.	MAKINGtheLINK program to overcome help-seeking barriers for MSUP in adolescents. Five interactive classroom activities run over 2 schools' periods, plus booster session, 11 schools, n = 1130.	Waitlist control, 11 schools, n = 1317.	Help-seeking (AHSQ), help-seeking barriers (BASH-B), likelihood and confidence in seeking professional help, and confidence to assist a peer to seek professional help, measured at baseline, 6-week post-intervention, and 6-months, and 12-months follow-ups.
Mankiewicz, 2024 and Tan, 2024 (SG)*[52,49]	RCT - Individual	168 participants recruited online, mean age = 20.7 (SD = 2.2, range = 18-26), 81.5% women, and 18.5% men.	90-second video of a woman sharing her experiences of, and recovery from, psychosis, n = 58.	Transcription of the intervention video, n = 55; and a 90-sec video featuring a woman sharing her experiences of surviving	Stigmatizing behavior (RIBS) and Attitudes towards mental illness (AMI-SG), measured at baseline, post-intervention, and 30-day follow-up.

© 2025 Crockett MA et al. *JAMA Network Open.*

				breast cancer, n = 55.	
Mann, 2008 (US)[41]	RCT - Cluster (classes)	53 undergraduate students in introductory psychology classes, mean age = 18.9 years old, 62.3% women, and 37.7% men.	Six-hour class focused on psychopathology, with first-person narratives and videos by people with mental illness. Participants wrote a poem from the perspective of a person with mental illness, n = 27.	Six-hour class focused on psychopathology, with clinician narratives and videos about mental illnesses. Participants conducted a diagnostic task, n = 26.	Social distance (SDS) measured at baseline and post-intervention.
Martin, 2022 (US)*[115]	RCT - Individual	1093 youth recruited from a crowdsourcing platform, mean age = 16.8 (SD = 1.1, range = 14-18), 45% female, 45% male, and 10% non-binary and prefer not to say.	117-second video of a Black girl who describes her difficulties coping with depressive symptoms and seeking help, n = 448.	A 117-sec video of a girl discussing aspects unique to being a Black adolescent girl and getting treatment, n = 456; and a 117-sec video of the same girl describing her hobbies, n = 189.	Stigma towards depression (DSS) and treatment-seeking intentions (GHSQ), measured at baseline and post-intervention.
Masuda, 2007 (US)*[59]	RCT - Individual	95 undergraduate college students, mean age = 19.7 years old, 67.4% female, 30.5% male, and 2.0% gender not specified.	Acceptance and commitment intervention, 150-min workshop, n = 52.	150-min educational workshop, n = 43.	Stigmatizing attitudes (CAMI) measured at baseline, post-intervention, and 1-month follow-up.
Milin, 2016 (CA)*[77]	RCT - Cluster (schools)	534 high school students from 24 schools, mean age = 16.5 years old (SD = 0.98), 55.1% female, and 44.9% male.	The Curriculum Guide has 6 modules about stigma of mental illness, understanding of mental health and mental illness, information on specific mental illnesses, experiences of mental illness, seeking help, and	TAU, 7 schools, n = 172.	Mental health knowledge and attitudes toward mental illness (made for study), measured at baseline and post-intervention.

© 2025 Crockett MA et al. *JAMA Network Open.*

			the importance of positive mental health, 6 hours of classroom time, 17 schools, n = 362.		
Morgan, 2019 (AU)*[116]	RCT - Individual	384 dyads of parents and adolescents, mean age = 13.3, (range 12–15), 55.6% female, and 44.4% male adolescents.	YMHFA: 4 face-to-face sessions (14-hours) to recognize the early signs of a mental health problem, to identify mental health-related crises, and to assist adolescents to get professional help, n = 51 parents.	PFA 15-hour training about knowledge and skills to sustain life, reduce pain and minimize the consequences of injury and illness until professional help arrives, n = 62 parents.	Quality of parental support, social distance (SDS), stigma, intended help-seeking, knowledge about mental health problems, mental health problem recognition, quality of MHFA intentions, confidence, quality of MHFA support, parent support towards adolescents, parent help-seeking, adolescent help-seeking. Most of the instruments were made for the study. Measured at baseline, and 12 and 24-months follow-ups.
Mori, 2022 (JP)*[66]	RCT - Individual	125 school students, age range = 12-13, 52.6% female, and 47.4% male.	Samita, a mental health literacy program that includes short-story animated films, filmed social contact, and educator's manuals. It was delivered in three 50-min classes, n = 51.	Control received three 50-min classes on healthy living and disease prevention, n = 74.	Knowledge of mental illness (MIDUS), attitudes towards people with mental health problems (RIBS-J), and help-seeking behaviour (made for study), measured at baseline, post-intervention, and 3-months follow-up.
Morrison, 2013 (UK)*[117]	RCT - Individual	288 young people with at-risk mental states, mean age = 20.7 years old (SD = 4.34; age	Cognitive therapy plus monitoring on a weekly basis for up to a maximum of 26 weeks, plus up to four booster sessions in the	Monitoring group (TAU), n = 144.	Internalized stigma (PBEQ) measured at baseline, 6, 12, 18, and 24-months follow-up.

© 2025 Crockett MA et al. *JAMA Network Open.*

		range = 14–35), 37.5% female, and 62.5% male.	subsequent six months, n =144.		
Mulfinger, 2017 (DE)[42]	RCT - Individual	98 adolescents with mental illness, mean age = 15.8-15.7 (SD = 1.2-1.1), 69% female, and 31% male.	Honest, Open, Proud is a three weekly 2-hour session peer-led group program that supports participants with disclosure decisions to reduce stigma's impact, plus TAU, n = 49.	TAU, n = 49.	Stigma stress scale, empowerment scale, attitudes toward disclosure (2 items), and self-stigma (ISMI and SSMIS), measured at baseline, post-intervention, and 3-weeks follow-up.
Norman, 2017 (CA)*[107]	RCT - Individual	218 (mostly) university students, mean age = 22.1 years old, 74.3% female, and 25.7% male.	Recovery-focused 10-min video regarding a person with schizophrenia with emphasis on his recovery (n = 73). Symptom-focused 10-min video in which acute phases of his illness were described (n = 72).	Photo and description of the same patient, n = 73.	Overall impression (made for study), perceived similarity to target (IOS), social distance, sympathy, and empathy for patient (made for study), measure at baseline, post-intervention, and 2-weeks follow-up.
O'Kearney, 2006 (AU)[43]	RCT - Individual	78 secondary students, age range = 15-16 years old, 100% males.	Mood GYM: self-paced interactive internet program that aims to help people identify problems with depression, to help overcome these problems, and to develop good coping skills. 5 main modules, 30–60 minutes each, n = 40.	Usual personal development program scheduled by the school for the 5 weeks of the trial, consisted of private study, ad hoc discussion and physical activities, n = 38.	Stigma towards depression (DSS), measured at baseline, post-intervention, and 16-weeks follow-up.
Oduguwa, 2017 (NG)*[78]	RCT - Cluster (wards)	205 students at five secondary schools from two administrative wards in Nigeria, mean age = 14.9 years	Mental health awareness training (total of 5 hours over 3 days) using didactic lectures, group discussions, and role plays to teach presentations, causes, and treatment of mental	Control condition (details not reported), 2 schools, 1 ward, n = 82.	Knowledge, attitudes, and social distance towards mental illness (UKPQ), measured at baseline, post-intervention, and 3-weeks follow-up.

© 2025 Crockett MA et al. *JAMA Network Open.*

		old, 47% female, and 53% male.	illness, 3 schools, 1 ward, n = 123.		
Ojio, 2020 (JP)*[103]	RCT - Individual	178 high schools and universities students, mean age = 21.9 years old, 55.3% female, and 44.7% male.	Biomedical messages group received a 10-min film covering biological mechanism of mental illnesses, pharmacological mechanism and gene-environment interaction, n = 90	Recommended messages video lecture group, 10-min film covered high prevalence of mental illnesses, recovery-oriented messages, and social inclusion/human rights messages, n = 89.	Knowledge of mental illness (MIDUS), past and future behavioral intentions (RIBS-J), help-seeking intention (single item), and intention to disclose (single item), measured at baseline, post-intervention, 1 and 12-month follow-up.
O'Mara, 2013 (CA)[44]	RCT – Cluster (classes)	294 school students from 6 schools (grades 9 and 10), 56.7% female, and 43.3% male.	75-min group discussion about mental health issues, n = 157.	Control group without intervention, n = 137.	Stigma (AQ), measured at baseline and 4-weeks follow-up.
Oshima, 2023 (JP)*[69]	RCT - Individual	49 adolescents diagnosed with autism and their caregivers, mean age = 13.5 (SD = 2.2, age range = 10-17), 26.7% female, and 73.3% male.	ACAT and TAU delivered in six weekly 100-min sessions. The first is a CBT intervention designed to increase the recipient's awareness of a child's autistic traits, to understand the child's autistic responses, and to practice problem solving skills, delivered by a therapist, n = 25.	TAU, n = 24.	Understanding of autistic attributes (AKQ-C), and barriers to care (BACE-3), measured at baseline, post-intervention, 10-weeks follow-up.
Papish, 2013 (CA)*[118]	RCT – Cluster (courses)	111 undergraduate medical students, 63.9% was between 18-25 years old, and 36.1% was 26-44 years old, 59.5% female, 40.5% male, and 13% had	Early one-time contact based educational intervention, where patients shared their story of having a mental illness, through two 1-hour presentations. A psychiatry course using the biopsychosocial model. Included patient presentation	Late intervention group received one-time contact based educational intervention at the end of a psychiatry course. Patients shared their story of having a mental illness, through	Stigma (OMS-HC), measured baseline, post-intervention, and 3-months follow-up.

© 2025 Crockett MA et al. *JAMA Network Open.*

		previous mental health treatment.	(2 hours) and “clinical correlations” sessions (6 hours) – small group teaching sessions, n = 55.	two 1-hour presentations. A psychiatry course using the biopsychosocial model. Included patient presentation (2 hours) and clinical correlations sessions (6 hours) – small group teaching sessions, n = 55.	
Patten, 2012 (CA)*[119]	RCT - Individual	211 3rd and 4th year undergraduate pharmacy students, mean age = 23.8 years old (SD = 2.6), 73,3% female, and 26.7% male.	Early contact-based education involved learning about mental illness from people with first-hand experience. One session delivered face to face for 1-2 hours, n = 58	Late contact-based education involved learning about mental illness from people with first-hand experience. One session delivered face to face during 1 to 2 hours, n = 73.	Stigma (OMS-HC), measured at baseline, post-intervention, and 1-month follow-up.
Penn, 2003 (US)*[79]	RCT - Individual	163 undergraduate students, mean age = 18.9 years old, 55.8% female, and 44.2% male.	Documentary about schizophrenia depicting various patients with the diagnosis in diverse daily settings, n = 38.	Documentary about polar bears (n = 41); documentary about weight concerns (n = 40); control group without intervention (n = 39).	Social distance (SDS), dangerousness (DS), negative emotions towards people with mental illness (AS), attributions about schizophrenia (made for study), behavioral intentions (IBI), measured post-intervention.
Perry, 2014 (AU)*[104]	RCT – Cluster (classes)	380 students from 10 schools, mean age = 14,8 years old, 50% female, and 50% male.	HeadStrong program contains five modules with information on mood disorders, activities to implement in their classrooms, and guidance on how to	Health and physical education classes, 5 schools, n = 173.	Depression literacy (D-Lit), personal stigma towards depression (DSS), and help-seeking attitudes (IASMHS), measured at

© 2025 Crockett MA et al. *JAMA Network Open.*

			deliver the activities, 5 schools, n = 207.		baseline, post-intervention, and 6-months follow-up.
Pinto-Foltz, 2011 (US)*[83]	RCT – Cluster (grades)	156 adolescents from 2 public high schools, mean age = 15 years old (range = 13-17), 100% females.	In Our Own Voice is a knowledge-contact intervention (narrative storytelling, discussion, and a video presentation) with five components presenting information using personal experience that considers both biological and environmental factors that may contribute to developing and recovering from mental illness, 1 60-min session, n = 95.	Control group without intervention, n = 61.	Mental illness stigma (AQ), and mental health literacy (made for study), measured at baseline, post-intervention, 4- and 8-weeks follow-up.
Pos, 2019 (NL)*[53]	RCT - Individua l	99 patients with early psychosis treatment, mean age = 25 years old, 19.2% female, 80.8% male, 20.3% had active delusions, 32.3% had active hallucination s with mild to moderate severity, and 15.5% with clinically relevant depressive symptoms.	CBTsa condition received eight group sessions of 1 hour/twice a week followed by six weekly individual sessions of 45 min. Treatment was delivered over a period of 3 months, n = 49.	TAU consisted of early intervention programs in which patient's symptoms, functioning and medication use were monitored for 3 years, n = 50.	Stigma (ISMI) measured at baseline, post-intervention, and 6-months follow-up.
Primbs, 2023 (DE)[45]	RCT - Individua l	50 adolescents with a history of depression, mean age = 15.8 (SD = 1.5, age	Information booklet about depression, n = 25.	Information booklet about asthma, n = 25.	Knowledge on depression (made for study), measured at baseline, post-intervention, and 4-weeks follow-up.

© 2025 Crockett MA et al. *JAMA Network Open.*

		range = 12-18), 72% female, and 28% male.			
Queroue, 2023 (FR)[46]	RCT - Individual	101 college students, median age = 20-21, 76.2% female, 23.8% male.	Interactive video about psychological distress among university students, recognizing depression symptoms, suicide risk warnings, and help-seeking, n = 50.	Brochures on depression and suicide risk, n = 51.	Mental health literacy (MHLS, The Mental Health & High School Curriculum Guide for students, MHPK-10) and help-seeking (GHSQ), measured at baseline and post-intervention.
Rahman, 1998 (PK)*[92]	RCT - Cluster (schools)	100 adolescents from 4 secondary schools at a rural subdistrict in Pakistan. Each student enlists into the study one of their parents, one friend, and one neighbor. Student's age range = 12-16 years old, 50% girls, and 50% boys.	School-mental health program incorporated into school curriculum using various participatory educational methods to improve understanding and awareness of mental disorders, 2 schools, n = 50.	Control condition (details not reported), 2 schools, n = 50.	Attitudes towards mental disorders (made for study), measured at baseline and post-intervention.
Rainone, 2018 (US)[47]	RCT - Individual	280 students from communication and information science departments, mean age = 20.44 years old, 74% female, 26% male, and 42% had a history of depression.	Antidepressant ads with portrayals of discrimination, cognitive separation, and stereotyping (n = 81); antidepressant ads without the latter (n = 90). Both conditions viewed three different antidepressant ads (2 min and 30 s total).	Control group without intervention, n = 109.	Social distance (RIBS), measured at baseline, and post-intervention.
Ranson, 2014 (AU)*[80]	RCT - Cluster (classes)	273 7th, 8th, and 9th grade students in an	Anti-stigma program consisting of 8 weekly 50-min	No-intervention peer condition	Knowledge (AKQ-R), attitudes (ACL),

© 2025 Crockett MA et al. *JAMA Network Open.*

		independent catholic school for children with special needs, range age = 12-15 years old, 100% females.	sessions combining education, direct (15-min) and video contact with individuals experiencing HFA, 2 classes, n = 48.	(n = 56); and no-intervention non-peer condition (n = 169).	and willingness to engage with people with HFA (SAQ-R), measured at baseline, 1-week post-intervention, and 3-months follow-up.
Reavley, 2014 (AU)*[120]	RCT – Cluster (campuses)	767 university students, mean age = 23.96-24.89 years old, 61.8% female, and 38.2% male.	Messages with information about mental disorders (depression) and sources or professional help delivered in diverse ways over 2 years, n = 426.	Control group without intervention, n = 341.	Help-seeking intentions, beliefs about the helpfulness of interventions, stigmatizing attitudes, help-seeking, mental health first aid given to family or friends, help-seeking intentions and actions taken for alcohol problems (made for study), measured at baseline and post-intervention.
Rubio-Valera, 2018 (ES)*[90]	RCT - Cluster (classes)	166 1st years social work students (8 classes), mean age = 21.9 years old, 89.2% female, 10.8% male, and 29.9% had previous experience with MI.	1-hour educational workshop led by an OBERTAMENT activist (people with a mental illness and psychoeducation training), 4 classes, n = 87	Waitlist control, 4 classes, n = 79	Intended behavior (RIBS), perceived and personal stigma (DSS), and stigmatizing attitudes (CAMI), measured at baseline, post-intervention, and 3-months follow-up.
Rusch, 2009 (US)*[96]	RCT - Individual	74 undergraduate psychology students, mean age = 21.45 years old, 72.1% female, and 27.9% male.	Contextual program: single 10-min session to discuss how depression is triggered by negative events (n = 23); biomedical program: single 10 min-session to discuss how depression is caused by chemical imbalances (n = 25).	Control program: 6-min single session addressing depression stigma but did not discuss the biomedical or contextual model of depression (n = 26); No-program control (n = 12).	Depression attribution (DAQ-27) and treatment seeking (made for study), measured at baseline, post-intervention, 1-week and 1-month follow-up.

© 2025 Crockett MA et al. *JAMA Network Open.*

Saif, 2019 (US)[48]	RCT - Individual	582 high school and college students, age range = 14-24 years old.	Celebrity webinar: interactive lessons providing evidence-based Alzheimer's disease (AD) education narrated by an actor (n high school students = 81; n college students = 51); Celebrity-video: video lessons providing evidence-based AD education narrated by an actor (n high school students = 90, n college students = 60); Interactive doctor webinar: lessons providing evidence-based AD education narrated by physician (n high school students = 113; n college students = 52).	Video lessons providing alternative AD content unrelated to study outcome, n high school students = 77, n college students = 58.	Knowledge about AD risk factors and willingness to make lifestyle changes, stigma toward people with AD, willingness to volunteer, and promote awareness/fundraise for AD (made for study), measured at baseline and post-intervention.
Sebastian, 2017 (AU)*[121]	RCT - Individual	245 psychology students, median age = 19 years old, 49% female, and 51% male.	Embodied conversation agent, 20-30 min sessions contact and education about anorexia nervosa, n = 123.	8-min video contact and video education about anorexia nervosa, n = 122.	Recognition of anorexia nervosa, positive and negative volitional stigma (made for study), and social distance (SDS), measured at baseline, and post-intervention 1 and post-intervention 2.
Sharp, 2006 (US)*[97]	RCT - Individual	123 undergraduate students from a large public university, mean age = 20 years old, 61% female, and 39% male.	Single-session lecture aimed at modifying mental health stigma and treatment fearfulness, and providing information on community resources, n = 62.	Astronomy science 40-min video, n = 61.	Attitudes towards mental illness (OMI), help-seeking attitudes (ATSPPHS-SF), and actual help-seeking (made for study) measured at baseline, post-intervention, and 4-weeks follow-up.
Simkiss, 2023 (UK)*[67]	RCT - Cluster	1926 school students, age range = 13-14, 55.3%	The Guide Cymru programme, 12-week mental health literacy programme,	TAU, 17 schools, n = 1863.	Knowledge and attitudes towards mental health (KAMHS),

© 2025 Crockett MA et al. *JAMA Network Open.*

		female, and 44.7% male.	12 schools, n = 1101.		measured at baseline and post-intervention.
Staniland, 2013 (AU)*[81]	RCT – Cluster (classes)	395 students from 17 schools (7th to 9th grade), mean age was not specified, 100% males.	Understanding Our Peers: an autism anti-stigma program that educates students about HFA, emphasizing similarities between themselves and peers, six weekly 50-min sessions, n = 46.	2 control groups, no intervention peer (n = 66) and no intervention non peer (n = 283), who attended their regular classes during the periods when the program was administered	Knowledge (AKQ), attitudes (ACL), and willingness to engage with peers with HFA (SAQ), perceived control (PRQ), measured at baseline, post intervention and 10-weeks follow-up.
Stanley, 2018 (US)*[122]	RCT - Individual	32 undergraduate students with untreated psychiatric disorders, mean age = 20.8 years old, (SD = 2.71, range = 18-28), 71.9% female, and 28.1% male.	CBM-HS, computer-based three separate intervention visits lasting 15 min each (45 min total), that occurred in one-week intervals, n = 17.	45-min psychoeducation session, n = 15.	Help-seeking self-stigma (SSOSH), perceived stigma and structural barriers (PS), and treatment utilization (made for study), measured at baseline, post-intervention, and 2-months follow-up.
Swartz, 2017 (US)*[123]	RCT – Cluster (schools)	6679 high school students from 66 secondary schools, age range = 14-15 years old, 51.6-50.8% female, and 49.2% male.	Adolescent Depression Awareness Program: program that provides education about depression in adolescents, attempting to minimize stigmatizing attitudes toward people with depression (3-hour duration), 27 schools, n = 3681.	Waitlist, 27 schools, n = 2998.	Depression literacy (ADKQ) and mental health stigma (RIBS), measured at baseline, 6-weeks post-intervention and 4-months follow-up.
Tay, 2022 (SG)*[49]	RCT - Individual	175 college students recruited from social media, age range = 18-24, 70.9%	Website with inspirational quotes and HOPE, a four-session (10-min each) mental health literacy and psychological	Website with inspirational quotes, n = 78.	Depression (D-Lit) and anxiety (A-Lit) literacy, and personal stigma (DSS), measured at baseline, post-

		female, and 29.1% male.	intervention for two weeks. It included quizzes, videos, and illustrations about mental health, n = 97.		intervention, two-months follow-up.
Taylor-Rodgers, 2014 (AU)[50]	RCT - Individual	67 university students, mean age = 21.9 years old, 74.6% female, and 25.4% male.	Websites with information on depression, anxiety, and suicide. Each website followed the same format: vignette of typical young person experiencing the mental health problem, description and symptoms, challenging stigmatizing views, treatment, and help options, three weeks long, n = 33.	Psychoeducation and general health wellbeing sessions (online informational programs with public domain information on health), three weeks long, n = 34.	Depression (D-Lit), anxiety (A-Lit), and suicide (Literacy of Suicide Scale) literacy; depression (DSS), anxiety (GASS) and suicide (SOSS) stigma, help-seeking attitudes (ATSPPH-SF) and intentions (GHSQ), measured at baseline and post-intervention.
Tuijnman, 2022 (NL)*[124]	RCT - Cluster	185 school students, mean age = 13.4 (SD = 0.7, age range = 12-15), 45.4% female, and 54.6% male.	Moving Stories, a game-based program on mental health literacy and social contact in digital and nondigital world (1-week duration), n = 99.	Control without intervention, n = 86.	Symptom recognition, first aid confidence, first aid intentions and skills, first aid behavior, beliefs about help, help-seeking behavior (made for study), help-seeking intentions (GHSQ), and stigma (DSS and SDS), measured at baseline, post-intervention, 3-month follow-up, and 6-month follow-up.
Vila-Badia, 2016 (ES)*[93]	RCT – Cluster (schools)	280 students from 4 secondary schools, age range = 14-18 years old.	20-min documentary film related to adolescent mental disorder aim to produce empathy with the students. Once finished, a brainstorming session of questions with specialized mental healthcare	Control group without intervention, n = 152.	Stigmatizing attitudes (CAMI), measured at baseline and 1-week post-intervention.

© 2025 Crockett MA et al. *JAMA Network Open.*

			staff was held, n = 128.		
Winkler, 2017 (CZ)*[60]	RCT – Cluster (schools)	499 students from 21 nursing high schools, mean age = 18.4 years old, 92.8% female, and 7.2% male.	Seminar: designed to reflect the current state of art in destigmatization of mental illness, 45-min (7 schools, n = 165). Short video: 3 video spots on stigma, 2-3 min (7 schools, N = 138).	Leaflet designed to address three myths related to people with mental illness, 7 schools, n = 196.	Stigmatizing attitudes (CAMI), future intended behavior (RIBS-J), measured at baseline, post-intervention, and 3-months follow-up.
Wood, 2006 (US)*[82]	RCT - Individual	114 undergraduate students, mean age = 21.4 years old, 76% female, 24% male, and 2% prefer not to say.	In Our Own Voice program consisted of an 11-minute video divided into segments, with the purpose of opening minds, change attitudes, and educating about what it means to have a mental illness, n = 57.	Presentation and video about careers in psychology, and an interactive discussion, n = 57.	Social distance (SDS), knowledge and attitudes towards people with mental illness (made for study), measured at baseline and post-intervention.
Wright, 2006 (AU)*[125]	Controlled Clinical Trial - Individual	1200 young people, age range = 12 - 25 years old.	Multimedia community awareness campaign that encourages help-seeking and provide professional help-seeking sources, n = 600	Control group without intervention, n = 600.	Recognition of depression and psychosis, barriers to help, self-identified depression, and first sought help in previous year (made for study), measured at baseline and 14-months post-intervention.
Yuen, 2021 (HK)*[54]	RCT - Individual	206 college students, mean age = 21.8 (SD = 5.0, age range =18-64), 55.3% female, and 44.7% male.	Immersive animation, a 10-min virtual reality intervention about the daily life and the stigma experienced as an animated story protagonist with mixed anxiety and depressive disorder, n = 72.	First-person audio narration of the intervention condition using virtual reality, n = 69; and a virtual reality video about planets, n = 69.	Public stigma (PSAS), measured at baseline, post-intervention and 1-week follow-up.

Notes: * Studies included in the meta-analysis. A-Lit = Anxiety Literacy Questionnaire. ACAT = Awareness and Care for My Autistic Traits. ACL = Adjective Checklists. AD = Alzheimer's Disease. ADKQ = Adolescent Depression Knowledge Questionnaire. AHSQ = Actual Help-Seeking Questionnaire. AKQ = Autism Knowledge Questionnaire. AKQ-C = Autism Knowledge Questionnaire-Child. AKQ-R = Autism Knowledge Questionnaire – Revised. AMI-SG = Attitudes to Mental Illness questionnaire Singapore version. APS = Attitudes to Psychiatry Scale. AQ = Attribution Questionnaire. AS = Affect Scale. ATP-30 = Attitudes Towards Psychiatry-30. ATSPPHS = Attitudes Towards Seeking Professional Psychological Help Scale. ATSPPHS-SF = Attitudes Towards Seeking Professional Psychological Help Scale - Short Form. AU =

© 2025 Crockett MA et al. *JAMA Network Open.*

Australia. BACE-3 = Barriers to Access to Care Evaluation Scale version 3. BAS = Biological Attribution Scale. BASH-B = Barriers to Adolescents Seeking Help Scale - Brief Version. Brief A-ADS = Brief Adolescent Attitudes towards Dementia Scale. BSSD = Bogardus Scale of Social Distance. CA = Canada. CAMI = Community Attitudes toward the Mentally Ill scale. CBM-HS = Cognitive Bias Modification for Help-seeking Stigma. CBTsa = CBT focused on social activation. CES-D = Center for Epidemiological Studies Depression Scale. CL = Chile. CN = China. CORE-GP = Clinical Outcomes in Routine Evaluation for the General Population. CZ = Czech Republic. DAQ-27 = Depression Attribution Questionnaire-27. D-DS = Discrimination-Devaluation Scale. DE = Germany. D-Lit = Depression Literacy Questionnaire. DS = Dangerousness Scale. DSD = Desired Social Distance. DSS = Depression Stigma Scale. EAPS-TM = Attitudes Scale of Healthcare Professional's toward People with a Diagnosis of Mental Illness (acronym in Spanish). EQ = Emotional Quotient Scale. ES = Spain. FABI = Self-report Inventory of Fear and Behavioural Intentions toward the Mentally Ill. FR = France. FTS = Forcing Treatment Scale. GASS = Generalised Anxiety Stigma Scale. GHSQ = General Help-seeking Questionnaire. GR = Greece. HFA = Higher-functioning autism. HK = Hong Kong. HSERK = Help-Seeking Encouragement-Related Knowledge Scale. HSWS = Help-Seeking Willingness Scale. IASMHS = Inventory of Attitudes towards Seeking Mental Health Services. IAT = Implicit Association Test. IOS = Inclusion of Others in Self Scale. ISCI = Intentions to Seek Counseling Inventory. ISMI = Internalized Stigma of Mental Illness Inventory-Short Form. JP = Japan. KAMHS = Knowledge and Attitudes to Mental Health Scales. KIDS = Kids Insight into Dementia Survey. KT = Knowledge Test. LSMT = Life Story Memory Test. MAKS = Mental Health Knowledge Schedule. MHFA = Mental Health First Aid. MHLq = Mental Health Literacy questionnaire. MHPK-10 = Mental Health-Promoting Knowledge. MIDUS = Mental Illness and Disorder Understanding Scale. MISS = Mental Illness Stigma Scale. MSUP = Mental and substance use problems. MY = Malaysia. NG = Nigeria. NL = Netherlands. OMI = Opinions about Mental Illness. OMS-HC = Opening Minds Survey for Health Care Providers. PBEQ = Personal Beliefs about Experiences Questionnaire. PBS = Psychological Blame Scale. PDDS = Perceived Devaluation-Discrimination Scale. PFA = Red Cross Provide First Aid. PK = Pakistan. PKS = Psychiatric Knowledge Scale. PRQ = Perceived Responsibility Questionnaire. PS = Perceived Stigma and Barriers to Care for Psychological Problems Scale. PSAS = Public Stigma and Acceptance Scale. PSS = Public Stigma Scale. PT = Portugal. RAs = Residential advisors. RCT = Randomized controlled trial. RIBS = Reported and Intended Behaviours Scale. RIBS-J = Japanese version of the Reported and Intended Behaviour Scale. RU = Russia. SAQ = Shared Activities Questionnaire. SAQ-R = Shared Activities Questionnaire – Revised. SD = Standard Deviation. SDS = Social Distance Scale. SG = Singapore. SOSS = Stigma of Suicide Scale. SSDS = Self-Stigma for Depression Scale. SSOSH = Self-Stigma of Seeking Help. SSRPH = Stigma Scale for Receiving Psychological Help. TAU = Treatment as usual. tMHFA = Teen Mental Heath First Aid. TW = Taiwan. UK = United Kingdom. UKPQ = UK Pinfold Questionnaire. US = United States. WSHS = Willingness to Seek Help Scale. YMHFA = Youth Mental Health First Aid.

© 2025 Crockett MA et al. *JAMA Network Open.*

eTable 2. Risk of Bias Within Studies

Study ID	Random sequence generation	Allocation concealment	Blinding of participants and personnel	Blinding of outcome assessment	Incomplete outcome data	Selective outcome reporting
Amsalem, 2022a (US)	Unclear	Unclear	Unclear	Unclear	Unclear	Low
Amsalem, 2022b (US)	Unclear	Unclear	Unclear	Unclear	Unclear	Low
Amsalem, 2023a (US)	Unclear	Unclear	Unclear	Unclear	Unclear	Low
Amsalem, 2023c (US)	Unclear	Unclear	Unclear	Unclear	Unclear	High
Boucher, 2014 (US)	Unclear	Unclear	High	High	Unclear	Low
Brown, 2010a (US)	Unclear	Unclear	High	High	Low	Low
Brown, 2010b (US)	Unclear	Unclear	High	High	Low	Low
Brown, 2017 (US)	Low	Unclear	Low	Low	Low	Low
Bruins, 2018 (NL)	Low	Low	Low	Low	Unclear	Low
Campbell, 2011 (UK)	Low	Low	High	High	High	Low
Campos, 2018 (PT)	Low	Unclear	High	High	High	Low
Casañas, 2022 (ES)	Low	Low	Low	Low	Low	Low
Chan, 2009 (CN)	Low	Unclear	High	High	High	Low
Cheetham, 2020 (AU)	Low	Unclear	High	High	High	Low
Chisholm, 2016 (UK)	Low	Low	Low	Low	Low	High
Chung, 2004 (CN)	High	High	High	Low	Low	Low
Chung, 2005 (CN)	Low	Unclear	High	High	Unclear	High
Chung, 2022 (HK)	Unclear	Unclear	Unclear	Unclear	Unclear	Low
Conceição, 2022 (PT)	Low	Unclear	High	Unclear	Low	High
Conceição, 2024 (PT)	Low	Low	High	Unclear	Low	High
Corrigan, 2007 (US)	Low	Unclear	High	High	Unclear	Low
Corrigan, 2010 (US)	Low	Unclear	High	Unclear	Unclear	Low
Costin, 2009 (AU)	Low	Low	Low	Low	Low	Low
Davies, 2018 (UK)	Low	Low	High	High	Low	Low
Dzemaili, 2023 (CH)	Low	Low	Unclear	Unclear	Low	Low

© 2025 Crockett MA et al. *JAMA Network Open.*

Economou, 2012 (GR)	Low	Unclear	High	High	Low	Unclear
Economou, 2014 (GR)	Low	Unclear	Low	Low	Unclear	Unclear
Esanu, 2016 (PT)	Unclear	Unclear	Unclear	Unclear	Low	Low
Farina, 2020 (UK)	Low	Unclear	High	High	High	Low
Fernandez, 2016 (MY)	High	Low	Unclear	Unclear	Low	Low
Finkelstein, 2008 (RU)	Low	Unclear	High	Low	Low	Low
Giannakopoulos, 2012 (GR)	Low	Unclear	High	High	Unclear	Low
Grandón, 2023 (CL)	Unclear	Low	High	Low	Low	Low
Greif-Green, 2020 (US)	Low	Low	Low	Low	Low	Low
Gulliver, 2012 (AU)	Low	Low	Unclear	Unclear	Unclear	Low
Han, 2006 (TW)	Low	Unclear	Unclear	High	High	Low
Han, 2014 (TW)	Unclear	Unclear	High	High	Low	Low
Han, 2018 (TW)	Low	Low	Low	Low	Low	Low
Han, 2023 (CN)	Low	Low	High	Low	Low	Low
Hart, 2018 and 2022 (AU)	Low	Low	High	Low	Low	Low
Howard, 2018 (AU)	Low	Low	Unclear	Low	Low	Low
Jorm, 2010 (AU)	Low	High	High	High	High	Low
Kerby, 2008 (UK)	Low	Low	High	High	Low	Low
King, 2015 (US)	Low	Low	Low	Low	High	Low
Kirschner, 2022 (US)	Unclear	Unclear	Unclear	Unclear	Low	Low
Koike, 2016 and Yamaguchi, 2019 (JP)	Low	Low	High	High	High	Low
Kosyluk, 2016 (US)	Low	Low	Low	Unclear	Low	Low
Kruger, 2022 (US)	Low	Unclear	Unclear	Unclear	Low	Low
Lannin, 2013 (US)	Low	Unclear	Unclear	High	Unclear	Low
Lincoln, 2008 (DE)	High	Unclear	High	High	Low	Low
Link, 2020 (US)	Low	Unclear	High	Unclear	Unclear	High
Lipson, 2014 (US)	Low	Unclear	High	High	Low	Low
Lubman, 2020 (AU)	Low	Low	High	High	Low	Low
Mankiewicz, 2024 and Tan, 2024 (SG)	Unclear	Unclear	Unclear	Unclear	Low	Low

Mann, 2008 (US)	Low	Low	Unclear	High	Unclear	Unclear
Martin, 2022 (US)	Unclear	Unclear	Unclear	Unclear	Low	Low
Masuda, 2007 (US)	Low	Unclear	High	High	High	Low
Milin, 2016 (CA)	Low	Low	High	High	Unclear	Low
Morgan, 2019 (AU)	Low	Low	High	Low	Low	Low
Mori, 2022 (JP)	Unclear	Low	High	Low	Low	Low
Morrison, 2013 (UK)	Low	Unclear	High	Low	Low	Low
Mulfinger, 2017 (DE)	Low	Low	High	High	Low	High
Norman, 2017 (CA)	Low	Unclear	High	High	High	Low
O'Kearney, 2006 (AU)	High	High	High	Unclear	Low	Low
Oduguwa, 2017 (NG)	Low	Unclear	High	High	Low	Low
Ojio, 2019 (JP)	Low	Low	Unclear	Low	Low	Low
O'Mara, 2013 (CA)	High	High	Unclear	Unclear	Low	Low
Oshima, 2023 (JP)	Low	Low	High	Low	Low	Low
Papish, 2013 (CA)	Unclear	Unclear	Unclear	High	High	Low
Patten, 2012 (CA)	High	High	High	Unclear	High	Low
Penn, 2003 (US)	Low	Unclear	Low	High	Low	Low
Perry, 2014 (AU)	Low	Low	High	Low	Unclear	Low
Pinto-Foltz, 2011 (US)	High	High	High	Unclear	Low	Low
Pos, 2019 (NL)	Low	Low	Low	Low	Low	Low
Primbs, 2023 (DE)	Low	Unclear	High	High	Low	High
Queroue, 2023 (FR)	Low	Unclear	Unclear	Unclear	Low	High
Rahman, 1998 (PK)	Low	Low	High	High	Low	Low
Rainone, 2017 (US)	Low	Unclear	High	High	Unclear	High
Ranson, 2014 (AU)	High	Low	High	High	Unclear	Low
Reavley, 2014 (AU)	Low	Low	Low	Low	Low	Low
Rubio-Valera, 2016 (ES)	Low	Low	High	High	Low	Low
Rusch, 2009 (US)	High	Unclear	High	Low	Unclear	Low
Saif, 2019 (US)	High	High	High	Unclear	Unclear	Low
Sebastian, 2017 (AU)	Low	Low	Low	Low	Low	Low

© 2025 Crockett MA et al. *JAMA Network Open.*

Sharp, 2006 (US)	Low	Unclear	High	High	Low	Low
Simkiss, 2023 (UK)	Low	Unclear	High	High	High	High
Staniland, 2013 (AU)	High	Unclear	High	High	Unclear	Low
Stanley, 2018 (US)	Low	Low	Low	Low	Low	Low
Swartz, 2017 (US)	Low	Unclear	Unclear	High	Low	Low
Tay, 2022 (SG)	Low	Low	High	Low	Low	Low
Taylor-Rodgers, 2014 (AU)	Low	High	High	Low	Low	Low
Tuijnman, 2022 (NL)	Unclear	Unclear	Unclear	Unclear	Low	Low
Vila-Badia, 2016 (ES)	Low	Unclear	High	High	Low	Low
Winkler, 2017 (CZ)	Low	High	High	High	High	Low
Wood, 2006 (US)	Low	Unclear	High	High	Unclear	Low
Wright, 2006 (AU)	Unclear	High	High	High	Unclear	Low
Yuen, 2021 (HK)	Low	Unclear	High	Low	Low	Low

Notes. AU = Australia. CA = Canada. CL = Chile. CN = China. CZ = Czech Republic. DE = Germany. ES = Spain. FR = France. GR = Greece. JP = Japan. MY = Malaysia. NG = Nigeria. NL = Netherlands. PK = Pakistan. PT = Portugal. RU = Russia. SG = Singapore. TW = Taiwan. UK = United Kingdom. US = United States.

© 2025 Crockett MA et al. *JAMA Network Open.*

eTable 3. Univariable Multilevel Meta-Regression Analyses of Selected Study Characteristics for Short-Term Effects of Interventions vs Controls on Stigma-Related Attitudes

**Variable**	***Estimates***	***95% CI***	τ_s^2	τ_g^2	***p***
Risk of bias			0.19	0.04	0.905
Low	0.35	-0.58 to 1.27			
Some concerns	0.54	-0.19 to 1.27			
High	**0.37**	**0.17 to 0.58**			
Intervention content			0.19	0.05	0.907
Education	**0.40**	**0.12 to 0.68**			
Mixed	**0.42**	**0.13 to 0.72**			
Other	0.04	-0.96 to 1.04			
Social contact	0.36	-0.03 to 0.76			
Intervention format			0.20	0.04	0.888
Mixed	0.34	-0.10 to 0.77			
Non-video-based	**0.43**	**0.16 to 0.71**			
Other	0.04	-0.97 to 1.05			
Video-based	0.38	-0.02 to 0.77			
Age			0.18	0.04	0.639
18 years or less	**0.43**	**0.15 to 0.72**			
More than 18 years old	**0.34**	**0.09 to 0.59**			

Notes.
95% CI, 95% confidence interval for *g*.
τ_s^2, variance between studies – after inclusion of covariate.
τ_g^2, variance between outcomes – after inclusion of covariate.
p, p-value of the test for subgroup differences.
Statistically significant estimates ($p < .05$) are marked in bold.
Analyses were conducted for 75 outcomes in 27 studies with a mean sample size of 230 individuals.
Estimates represent the effect sizes (i.e., Hedge's *g*) for each category.

© 2025 Crockett MA et al. *JAMA Network Open.*

eTable 4. Univariable Multilevel Meta-Regression Analyses of Selected Study Characteristics for Short-Term Effects of Interventions vs Controls on Stigma-Related Knowledge

Variable	*Estimates*	*95% CI*	τ_s^2	τ_g^2	*p*
Risk of bias			0.22	0.06	0.773
Low	0.54	-0.43 to 1.51			
Some concerns	0.43	-0.31 to 1.18			
High	**0.70**	**0.44 to 0.96**			
Intervention content			0.23	0.05	0.169
Education	**0.48**	**0.18 to 0.79**			
Mixed	**0.73**	**0.40 to 1.06**			
Other	**0.71**	**0.11 to 1.32**			
Social contact	**0.88**	**0.45 to 1.31**			
Intervention format			0.21	0.06	0.144
Mixed	**0.83**	**0.39 to 1.28**			
Non-video-based	**0.44**	**0.11 to 0.76**			
Other	**0.70**	**0.12 to 1.29**			
Video-based	**0.87**	**0.46 to 1.29**			
Age			0.21	0.06	0.878
18 years or less	**0.64**	**0.32 to 0.97**			
More than 18 years old	**0.68**	**0.35 to 1.01**			

Notes.
ref., reference category.
95% CI, 95% confidence interval for *g*.
τ_s^2, variance between studies – after inclusion of covariate.
τ_g^2, variance between outcomes – after inclusion of covariate.
p, p-value of the test for subgroup differences.
Statistically significant estimates ($p < .05$) are marked in bold.
Analyses were conducted for 74 outcomes in 20 studies with a mean sample size of 352.5 individuals.
In this subgroup of analyses, there were no studies with a low risk of bias.
Estimates represent the effect sizes (i.e., Hedge's *g*) for each category.

© 2025 Crockett MA et al. *JAMA Network Open.*

eTable 5. Univariable Multilevel Meta-Regression Analyses of Selected Study Characteristics for Short-Term Effects of Interventions vs Controls on General Stigma

**Variable**	***Estimates***	***95% CI***	τ_s^2	τ_g^2	***p***
Risk of bias			0.04	0.01	0.640
Low	0.40	-0.27 to 1.06			
Some concerns	0.34	-0.09 to 0.77			
High	**0.17**	**0.00 to 0.34**			
Intervention content			0.03	0.01	0.372
Education	**0.34**	**0.07 to 0.61**			
Mixed	0.03	-0.22 to 0.29			
Other	0.40	-0.24 to 1.03			
Social contact	**0.23**	**0.03 to 0.43**			
Intervention format			0.06	0.01	0.689
Mixed	0.05	-0.35 to 0.44			
Non-video-based	0.32	-0.00 to 0.65			
Other	0.40	-0.33 to 1.12			
Video-based	0.21	-0.05 to 0.47			
Age			0.02	0.01	0.068
18 years or less	0.08	-0.09 to 0.26			
More than 18 years old	**0.32**	**0.13 to 0.52**			

Notes.
95% CI, 95% confidence interval for *g*.
τ_s^2, variance between studies – after inclusion of covariate.
τ_g^2, variance between outcomes – after inclusion of covariate.
p, p-value of the test for subgroup differences.
Statistically significant estimates ($p < .05$) are marked in bold.
Analyses were conducted for 30 outcomes in 13 studies with a mean sample size of 462.3 individuals.
Estimates represent the effect sizes (i.e., Hedge's *g*) for each category.

© 2025 Crockett MA et al. *JAMA Network Open.*

eTable 6. Univariable Multilevel Meta-Regression Analyses of Selected Study Characteristics for Short-Term Effects of Interventions vs Controls Stigma-Related Behaviors

**Variable**	***Estimates***	***95% CI***	τ_s^2	τ_g^2	p
Risk of bias			0.12	0.01	0.853
Low	0.16	-0.41 to 0.51			
Some concerns	0.26	-0.19 to 0.71			
High	**0.32**	**0.13 to 0.51**			
Intervention content			0.11	0.01	0.173
Education	**0.28**	**0.08 to 0.47**			
Mixed	**0.25**	**0.03 to 0.46**			
Other	0.01	-0.70 to 0.71			
Social contact	**0.43**	**0.22 to 0.64**			
Intervention format			0.13	0.01	0.234
Mixed	0.27	-0.05 to 0.59			
Non-video-based	**0.26**	**0.03 to 0.49**			
Other	0.01	-0.74 to 0.76			
Video-based	**0.43**	**0.19 to 0.67**			
Age			0.11	0.01	0.531
18 years or less	0.22	-0.05 to 0.50			
More than 18 years old	**0.33**	**0.13 to 0.53**			

Notes.
95% CI, 95% confidence interval for *g*.
τ_s^2, variance between studies – after inclusion of covariate.
τ_g^2, variance between outcomes – after inclusion of covariate.
p, p-value of the test for subgroup differences.
Statistically significant estimates ($p < .05$) are marked in bold.
Analyses were conducted for 66 outcomes in 22 studies with a mean sample size of 226.1 individuals.
Estimates represent the effect sizes (i.e., Hedge's *g*) for each category.
There were statistically significant subgroup differences for intervention content. Specifically, estimates from interventions based on social contact had a higher Hedge's *g* than those based on education (β = 0.15, 95% CI = 0.00 to 0.30, *p*-value = 0.045).

© 2025 Crockett MA et al. *JAMA Network Open.*

eTable 7. Univariable Multilevel Meta-Regression Analyses of Selected Study Characteristics for Short-Term Effects Between Active Interventions Stigma-Related Attitudes

Variable	*Estimates*	*95% CI*	τ_s^2	τ_g^2	*p*
1st intervention content			0.19	0.22	0.984
Education	0.27	-0.27 to 0.80			
Mixed	0.38	-0.38 to 1.14			
Other	0.45	-0.45 to 1.34			
Social contact	0.29	-0.26 to 0.84			
2nd intervention content			0.11	0.22	0.306
Education	0.28	-0.09 to 0.65			
Mixed	0.04	-0.46 to 0.54			
Other	**0.53**	**0.05 to 1.02**			
Social contact	0.25	-0.35 to 0.85			
1st intervention format			0.14	0.23	0.708
Mixed	-0.09	-0.97 to 0.79			
Non-video-based	**0.42**	**0.00 to 0.84**			
Other	0.45	-0.39 to 1.29			
Video-based	0.23	-0.22 to 0.69			
2nd intervention format			0.10	0.22	0.350
Mixed	0.24	-0.39 to 0.87			
Non-video-based	0.63	-0.73 to 1.99			
Other	**0.49**	**0.12 to 0.87**			
Video-based	0.18	-0.11 to 0.48			
Age			0.12	0.22	0.330
18 years or less	-0.09	-0.93 to 0.75			
More than 18 years old	**0.35**	**0.05 to 0.64**			

Notes.
ref., reference category.
95% CI, 95% confidence interval for *g*.
τ_s^2, variance between studies – after inclusion of covariate.
τ_g^2, variance between outcomes – after inclusion of covariate.
p, p-value of the test for subgroup differences.
Statistically significant estimates ($p < .05$) are marked in bold.
Analyses were conducted for 59 outcomes in 12 studies with a mean sample size of 117.5 individuals.
These analyses compare two active interventions (i.e., 1st vs. 2nd).
In this subgroup of analyses, all included studies had a high risk of bias.
Estimates represent the effect sizes (i.e., Hedge's *g*) for each category.

© 2025 Crockett MA et al. *JAMA Network Open.*

eTable 8. Univariable Multilevel Meta-Regression Analyses of Selected Study Characteristics for Short-Term Effects Between Active Interventions Stigma-Related Behaviors

Variable	*Estimates*	*95% CI*	τ_s^2	τ_g^2	*p*
Risk of bias			0.17	0.07	0.411
Some concerns	-0.03	-0.57 to 0.51			
High	0.22	-0.06 to 0.50			
1st intervention content			0.17	0.07	0.342
Education	0.07	-0.41 to 0.54			
Mixed	-0.16	-0.72 to 0.39			
Other	-0.02	-0.99 to 0.96			
Social contact	**0.40**	**0.04 to 0.77**			
2nd intervention content			0.11	0.07	0.062
Social contact	0.27	-0.24 to 0.78			
Education	0.16	-0.10 to 0.42			
Mixed	-0.25	-0.63 to 0.13			
Other	**0.43**	**0.02 to 0.85**			
1st intervention format			0.21	0.07	0.953
Mixed	-0.01	-1.00 to 0.98			
Non-video-based	0.19	-0.18 to 0.56			
Other	-0.02	-1.07 to 1.04			
Video-based	0.22	-0.13 to 0.57			
2nd intervention format			0.05	0.07	**0.003**
Video-based	0.08	-0.11 to 0.28			
Mixed	-0.35	-0.75 to 0.05			
Other	**0.33**	**0.06 to 0.60**			
Non-video-based	**1.62**	**0.51 to 2.74**			
Age			0.14	0.07	0.314
18 years or less	-0.06	-0.56 to 0.44			
More than 18 years old	0.23	-0.02 to 0.48			

Notes.
ref., reference category.
95% CI, 95% confidence interval for *g*.
τ_s^2, variance between studies – after inclusion of covariate.
τ_g^2, variance between outcomes – after inclusion of covariate.
p, p-value of the test for subgroup differences.
Statistically significant estimates ($p < .05$) are marked in bold.
Analyses were conducted for 52 outcomes in 15 studies with a mean sample size of 176.3 individuals.
These analyses compare two active interventions (i.e., 1st vs. 2nd).
Estimates represent the effect sizes (i.e., Hedge's *g*) for each category.
There were statistically significant subgroup differences for intervention format in the 2nd intervention group. Specifically, estimates from the 2nd intervention group based on a mixture of format had a lower Hedge's *g* than those based on video (β = -0.43, 95% CI = -0.83 to -0.04, *p*-value = 0.032). On the other hand, estimates from the 2nd intervention group based on a non-video-based format had a higher Hedge's *g* than those based on video (β = 1.54, 95% CI = 0.41 to 2.67, *p*-value = 0.008).

© 2025 Crockett MA et al. *JAMA Network Open.*

eTable 9. Univariable Multilevel Meta-Regression Analyses of Selected Study Characteristics for Short-Term Effects of Interventions vs Controls on Help-Seeking Intentions

**Variable**	***Estimates***	***95% CI***	τ_s^2	τ_g^2	***p***
Risk of bias			0.00	0.01	0.699
Low	0.02	-0.29 to 0.34			
Some concerns	0.21	-0.11 to 0.53			
High	**0.14**	**0.07 to 0.21**			
Intervention content			0.00	0.01	0.434
Education	0.15	-0.01 to 0.31			
Mixed	**0.21**	**0.07 to 0.34**			
Social contact	**0.11**	**0.03 to 0.19**			
Intervention format			0.00	0.01	0.497
Mixed	**0.21**	**0.07 to 0.34**			
Non-video-based	0.13	-0.05 to 0.31			
Video-based	**0.11**	**0.03 to 0.20**			
Age			0.00	0.01	0.352
18 years or less	**0.12**	**0.05 to 0.20**			
More than 18 years old	**0.21**	**0.04 to 0.38**			

Notes.
95% CI, 95% confidence interval for *g*.
τ_s^2, variance between studies – after inclusion of covariate.
τ_g^2, variance between outcomes – after inclusion of covariate.
p, p-value of the test for subgroup differences.
Statistically significant estimates ($p < .05$) are marked in bold.
Analyses were conducted for 110 outcomes in 10 studies with a mean sample size of 435.9 individuals.
Estimates represent the effect sizes (i.e., Hedge's *g*) for each category.
In this subgroup of analyses, no study used interventions having other contents, nor formats.

eTable 10. Meta-Analytic Estimates for Secondary Outcomes (Continuous Data)

Timing	Outcome	Intervention	*i*	*j*	$\bar{N}$	*g*	*95% CI*	*p*	τ_s^2	τ_g^2	I_s^2	I_g^2
Short-term	Helping behaviors[65,88,94,124]	Active vs. Control	4	15	475	-1.59	-5.70 to 2.53	.423	0.00	54.90	0.0%	100.0%
Short-term [a]	Helping behaviors[102]	Active vs. Active	1	3	139	0.28	-0.39 to 0.95	.217	0.00	0.00	0.0%	0.0%
Short-term [a]	Empowerment[106]	Active vs. Active	1	2	244	-0.72	-3.84 to 2.41	.210	-	0.09	-	83.7%

Notes.

i, number of studies.

j, number of outcomes.

$\bar{N}$, mean sample size.

d, mean Hedge's *g*.

95% CI, 95% confidence interval for *g*.

τ_s^2, variance between studies.

τ_g^2, variance between outcomes.

I_s^2, heterogeneity between studies.

I_g^2, heterogeneity between outcomes.

[a], single-level, multivariate meta-analysis.

Statistically significant estimates ($p < .05$) are marked in bold.

© 2025 Crockett MA et al. *JAMA Network Open.*

eTable 11. Meta-Analytic Estimates for Secondary Outcomes (Dichotomous Data)

Timing	Outcome	Intervention	*i*	*j*	$\bar{N}$	*OR*	*95% CI*	*p*	τ_s^2	τ_g^2	I_s^2	I_g^2
Short-term	Helping behaviors[65,114,120]	Active vs. Control	3	5	593.6	2.04	0.67 to 6.24	.134	0.39	0.00	85.2%	0.0%
Mid-term [a]	Helping behaviors[114]	Active vs. Control	1	2	1042	1.27	0.14 to 11.24	.398	-	0.03	-	62.9%
Long-term	Helping behaviors[65,114,109]	Active vs. Control	3	6	655.3	1.43	1.01 to 2.02	.341	0.00	0.03	0.0%	45.5%

Notes.
i, number of studies.
j, number of outcomes.
$\bar{N}$, mean sample size.
OR, Odds Ratio.
95% CI, 95% confidence interval for *OR*.
τ_s^2, variance between studies.
τ_g^2, variance between outcomes.
I_s^2, heterogeneity between studies.
I_g^2, heterogeneity between outcomes.
[a], single-level, multivariate meta-analysis.
Statistically significant estimates ($p < .05$) are marked in bold.

© 2025 Crockett MA et al. *JAMA Network Open.*

eFigure 1. Forest Plot of Short-Term Effects of Interventions vs Controls on Stigma-Related Attitudes

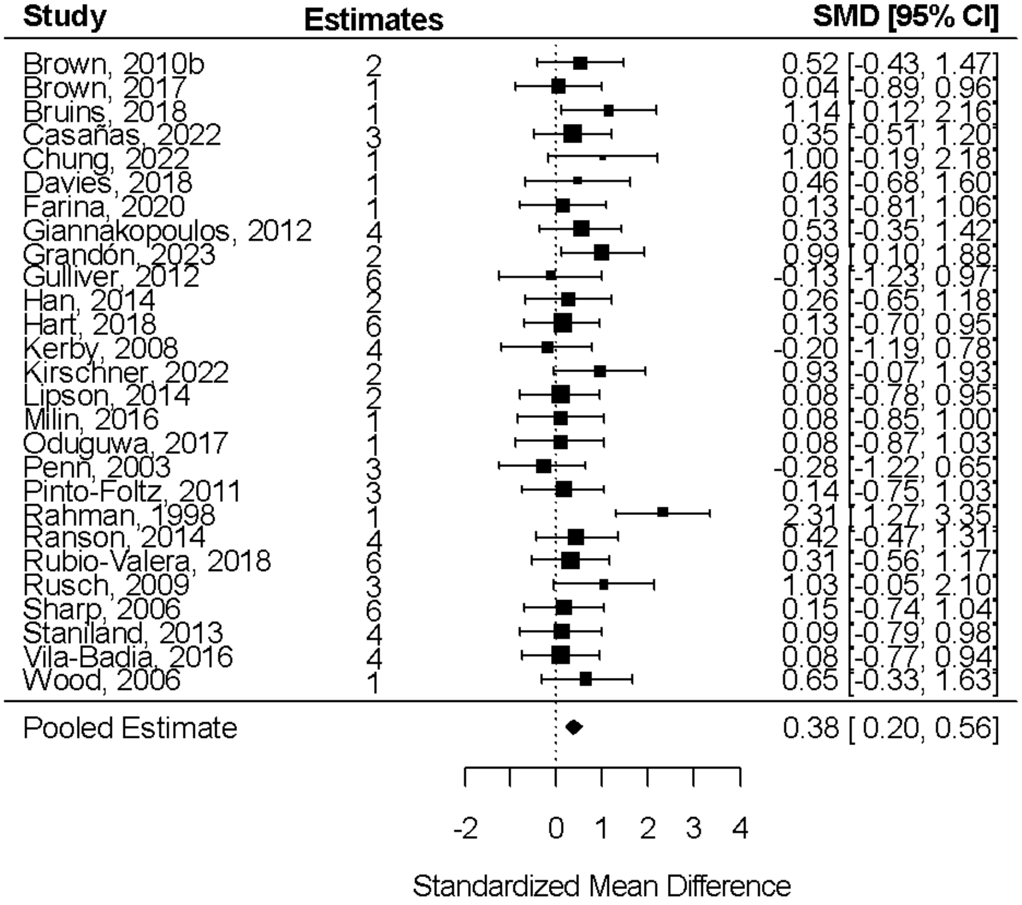

Notes.
Estimates were aggregated to the study level.
SMD, standardized mean difference (i.e., Hedge's *g*).
95% CI, 95% confidence interval.

© 2025 Crockett MA et al. *JAMA Network Open.*

eFigure 2. Forest Plot of Short-Term Effects of Interventions vs Controls on Stigma-Related Behaviors

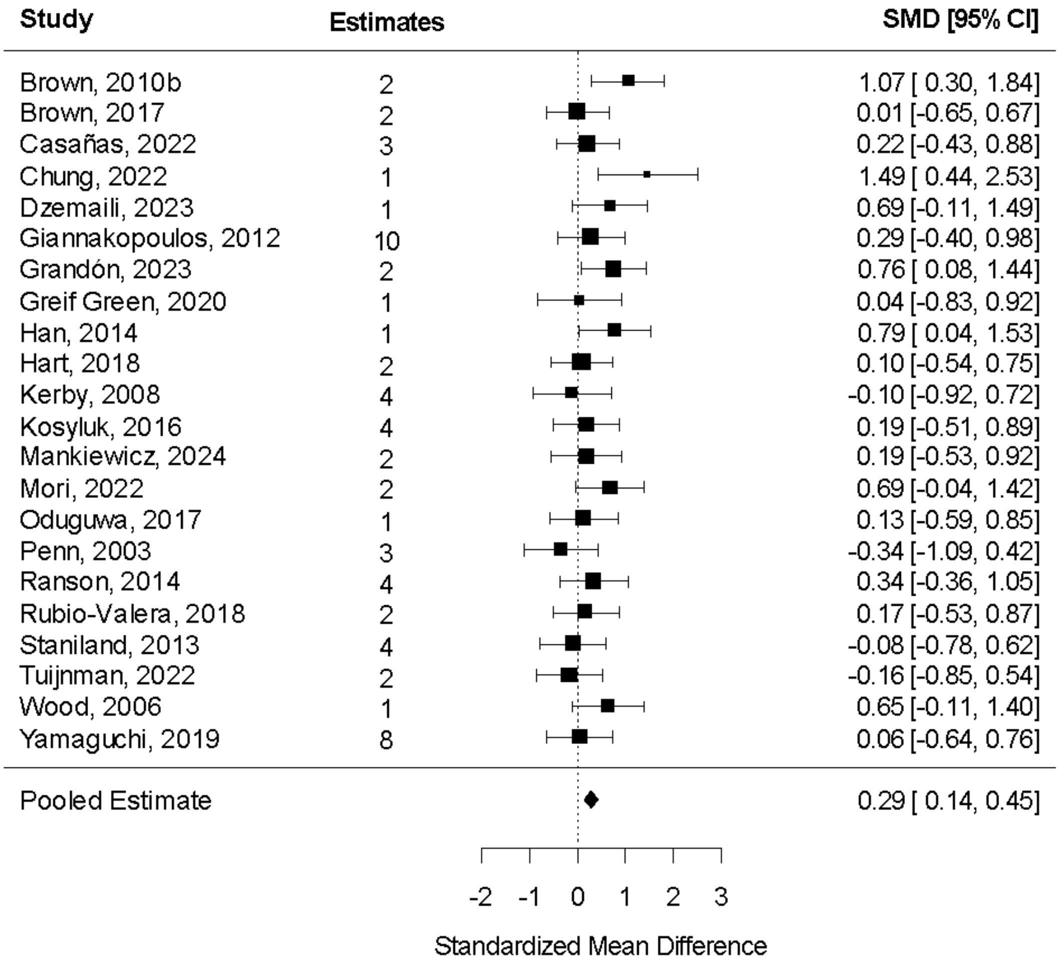

Notes.
Estimates were aggregated to the study level.
SMD, standardized mean difference (i.e., Hedge's *g*).
95% CI, 95% confidence interval.

© 2025 Crockett MA et al. *JAMA Network Open.*

eFigure 3. Forest Plot of Short-Term Effects of Interventions vs Controls on Stigma-Related Knowledge

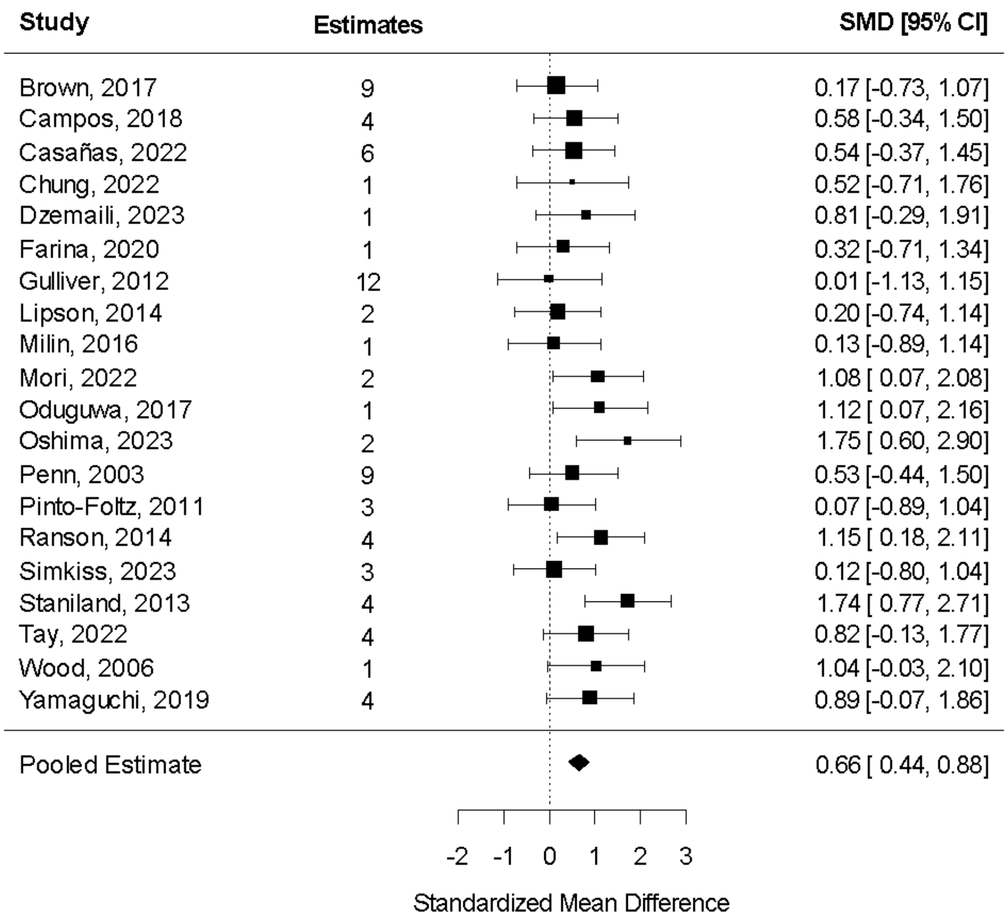

Notes.
Estimates were aggregated to the study level.
SMD, standardized mean difference (i.e., Hedge's *g*).
95% CI, 95% confidence interval.

© 2025 Crockett MA et al. *JAMA Network Open.*

eFigure 4. Forest Plot of Short-Term Effects of Interventions vs Controls on General Stigma

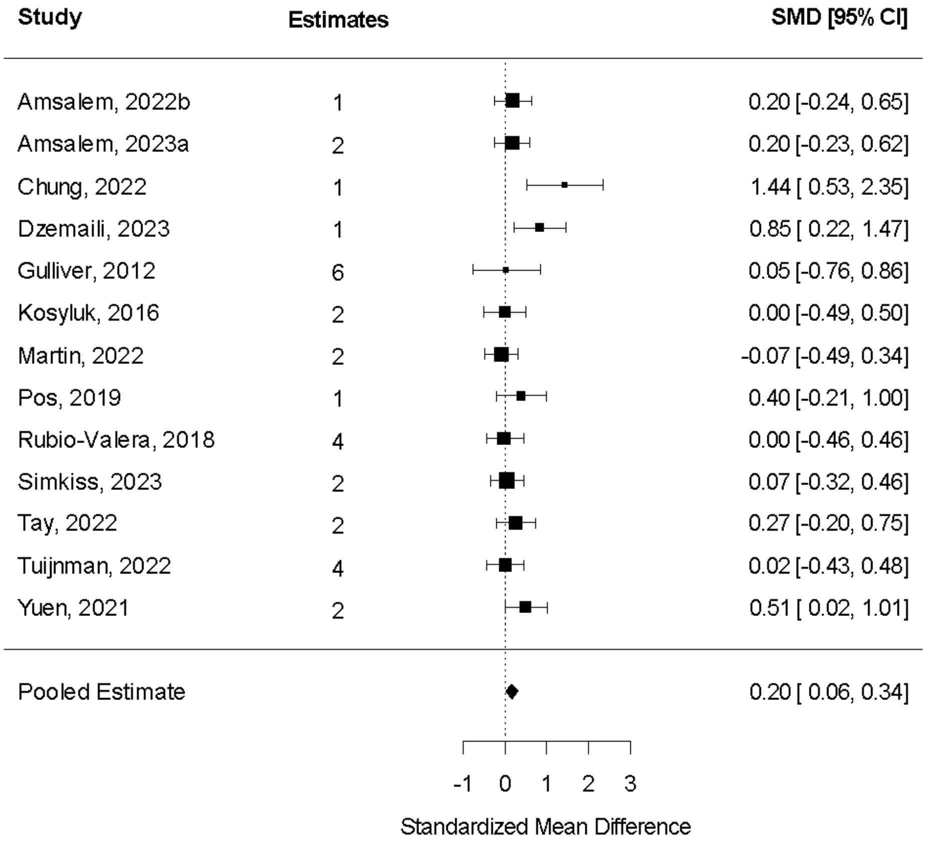

Notes.
Estimates were aggregated to the study level.
SMD, standardized mean difference (i.e., Hedge's *g*).
95% CI, 95% confidence interval.

© 2025 Crockett MA et al. *JAMA Network Open.*

eFigure 5. Forest Plot of Short-Term Effects Between Active Interventions on Stigma-Related Attitudes

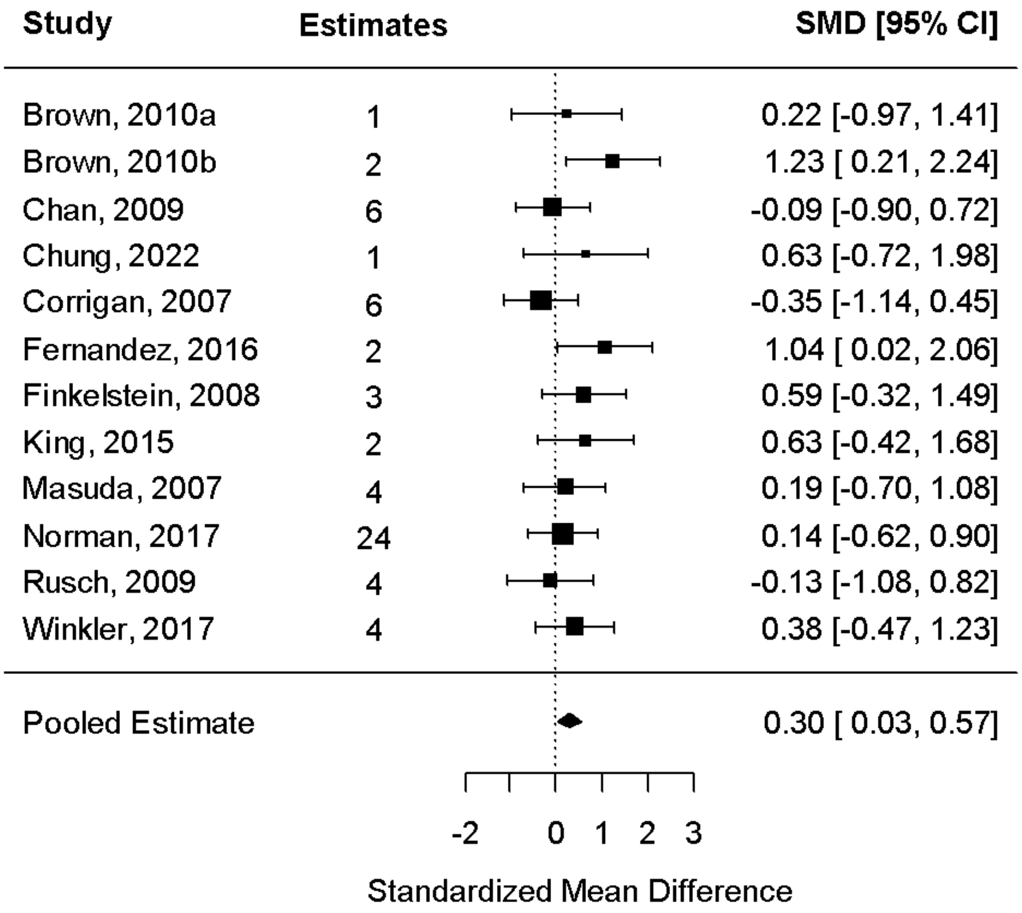

Notes.
Estimates were aggregated to the study level.
SMD, standardized mean difference (i.e., Hedge's *g*).
95% CI, 95% confidence interval.

© 2025 Crockett MA et al. *JAMA Network Open.*

eFigure 6. Forest Plot of Short-Term Effects Between Active Interventions on Stigma-Related Behaviors

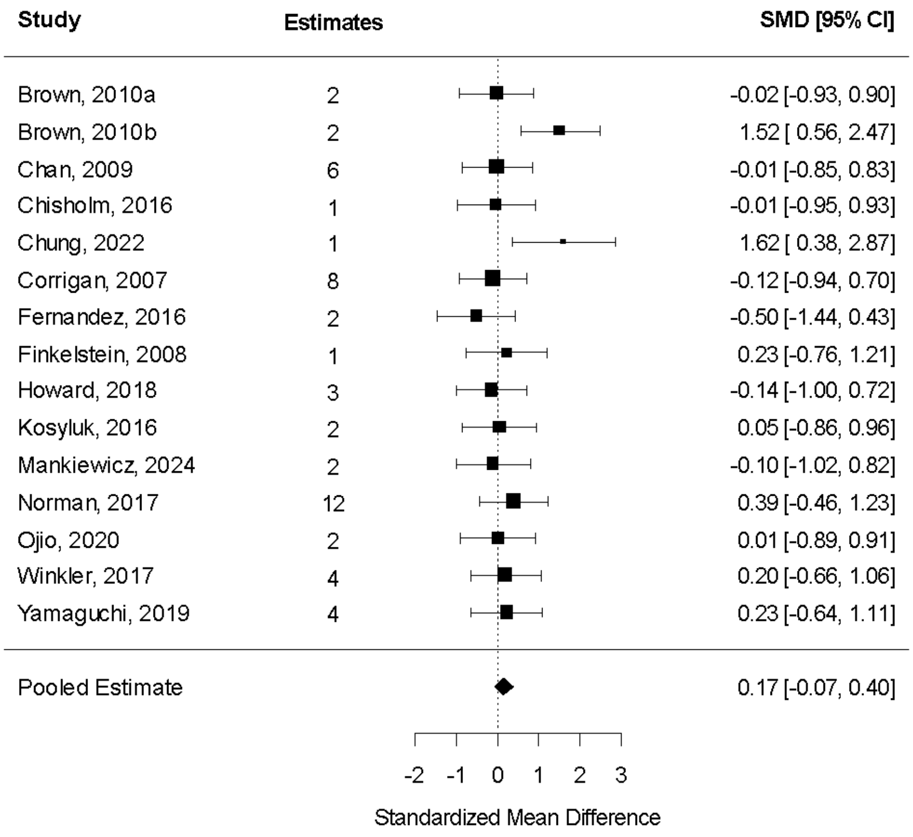

Notes.
Estimates were aggregated to the study level.
SMD, standardized mean difference (i.e., Hedge's *g*).
95% CI, 95% confidence interval.

© 2025 Crockett MA et al. *JAMA Network Open.*

eFigure 7. Forest Plot of Short-Term Effects of Interventions vs Controls on Help-Seeking Intentions

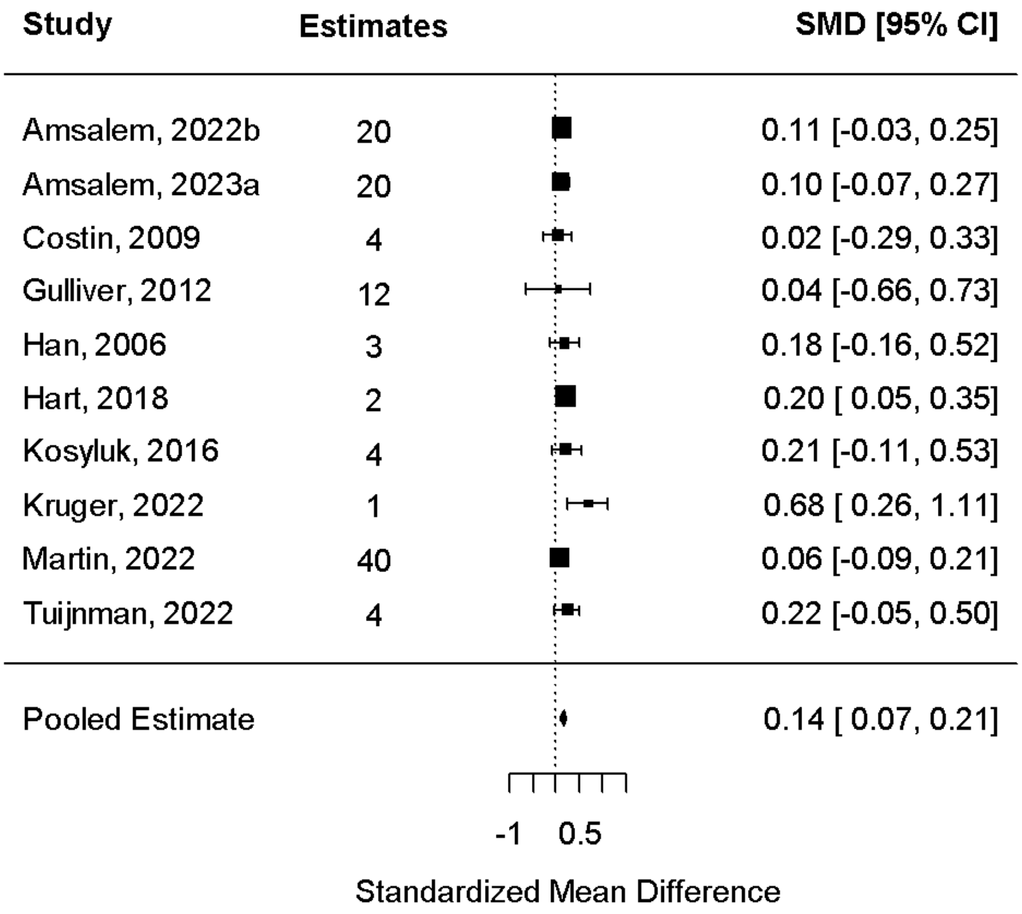

Notes.
Estimates were aggregated to the study level.
SMD, standardized mean difference (i.e., Hedge's *g*).
95% CI, 95% confidence interval.

© 2025 Crockett MA et al. *JAMA Network Open.*

eFigure 8. Funnel Plot of Short-Term Effects of Interventions vs Controls on Stigma-Related Attitudes

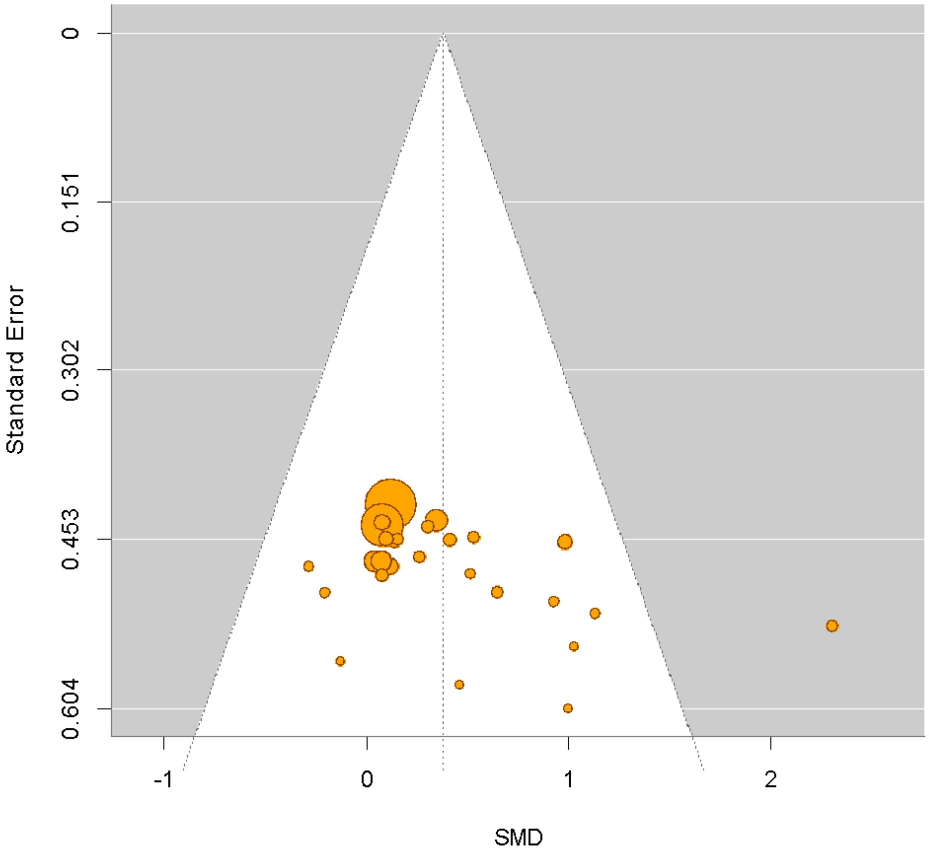

Notes.
Estimates were aggregated to the study level.
The plotted dots were weighted by the number of estimates per study.
SMD, standardized mean difference (i.e., Hedge's *g*).
In a multilevel meta-regression, the effect sizes' standard errors were included as the only moderator. The coefficient for the standard errors was statistically significant (β = 2.02, 95% CI = 0.18 to 3.86, *p*-value = 0.032). Therefore, there was evidence of asymmetry in the distribution of effect sizes.

© 2025 Crockett MA et al. *JAMA Network Open.*

eFigure 9. Funnel Plot of Short-Term Effects of Interventions vs Controls on Stigma-Related Behaviors

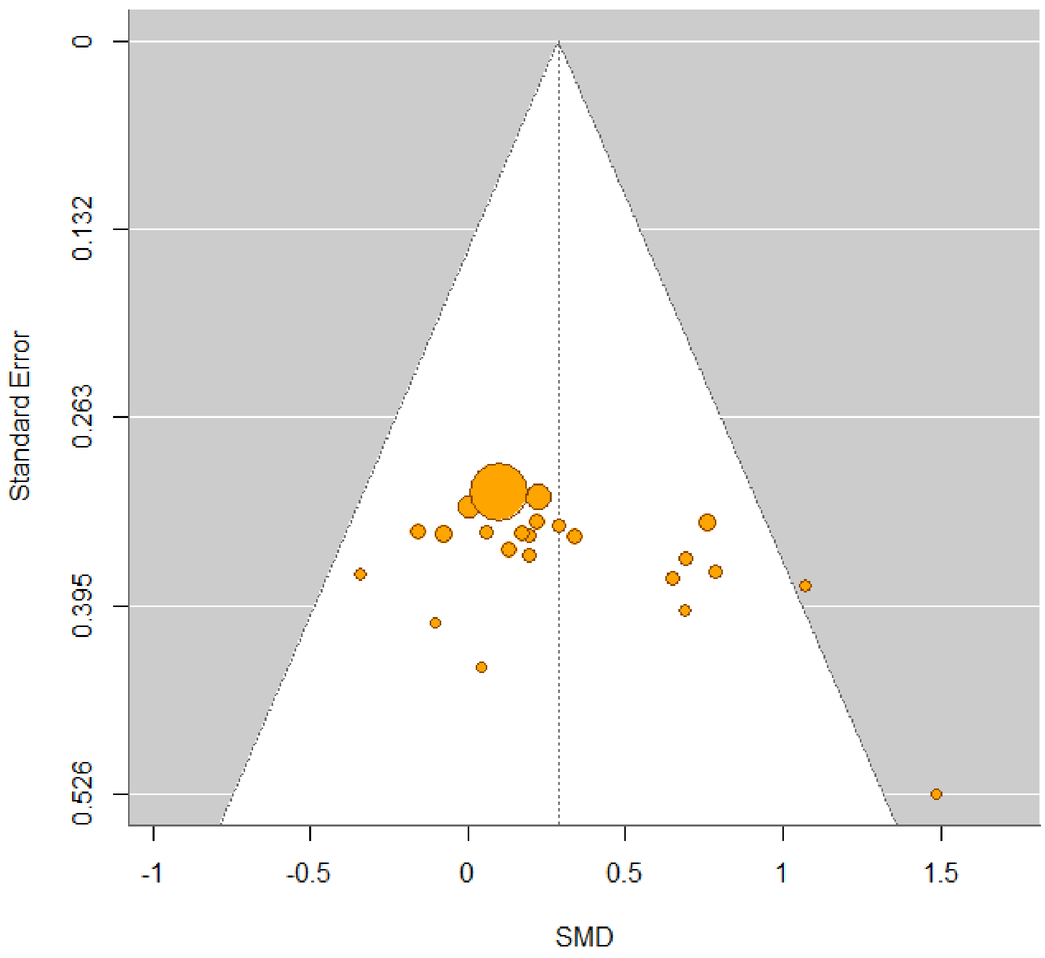

Notes.
Estimates were aggregated to the study level.
The plotted dots were weighted by the number of estimates per study.
SMD, standardized mean difference (i.e., Hedge's *g*).
In a multilevel meta-regression, the effect sizes' standard errors were included as the only moderator. The coefficient for the standard errors was not statistically significant (β = 1.64, 95% CI = -0.45 to 3.73, *p*-value = 0.122). Therefore, there was no evidence of asymmetry in the distribution of effect sizes.

© 2025 Crockett MA et al. *JAMA Network Open.*

eFigure 10. Funnel Plot of Short-Term Effects of Interventions vs Controls on Stigma-Related Knowledge

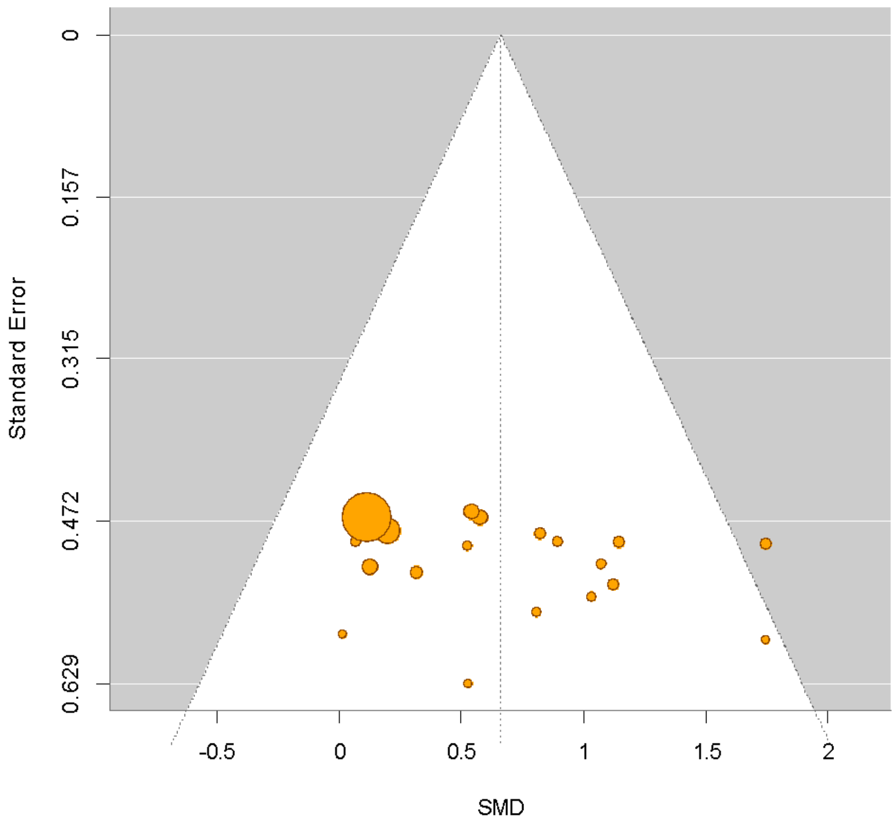

Notes.
Estimates were aggregated to the study level.
The plotted dots were weighted by the number of estimates per study.
SMD, standardized mean difference (i.e., Hedge's *g*).
In a multilevel meta-regression, the effect sizes' standard errors were included as the only moderator. The coefficient for the standard errors was statistically significant (β = 2.56, 95% CI = 0.47 to 4.65, *p*-value = 0.017). Therefore, there was evidence of asymmetry in the distribution of effect sizes.

© 2025 Crockett MA et al. *JAMA Network Open.*

eFigure 11. Funnel Plot of Short-Term Effects of Interventions vs Controls on General Stigma

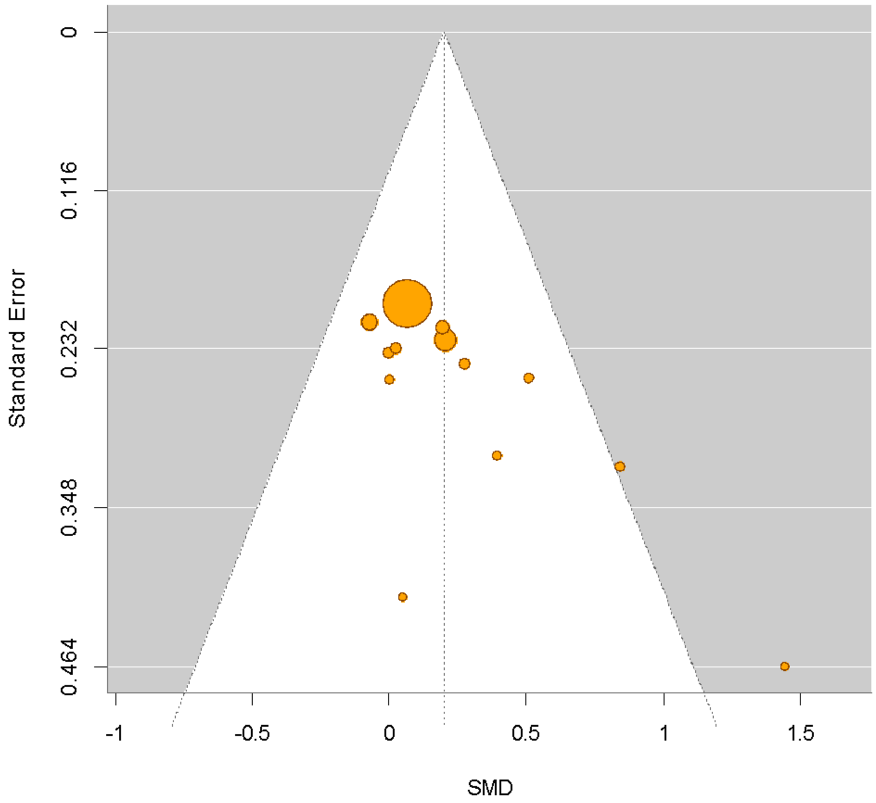

Notes.
Estimates were aggregated to the study level.
The plotted dots were weighted by the number of estimates per study.
SMD, standardized mean difference (i.e., Hedge's *g*).
In a multilevel meta-regression, the effect sizes' standard errors were included as the only moderator. The coefficient for the standard errors was statistically significant (β = 1.77, 95% CI = 0.29 to 3.25, *p*-value = 0.021). Therefore, there was evidence of asymmetry in the distribution of effect sizes.

© 2025 Crockett MA et al. *JAMA Network Open.*

eFigure 12. Funnel Plot of Short-Term Effects Between Active Interventions on Stigma-Related Attitudes

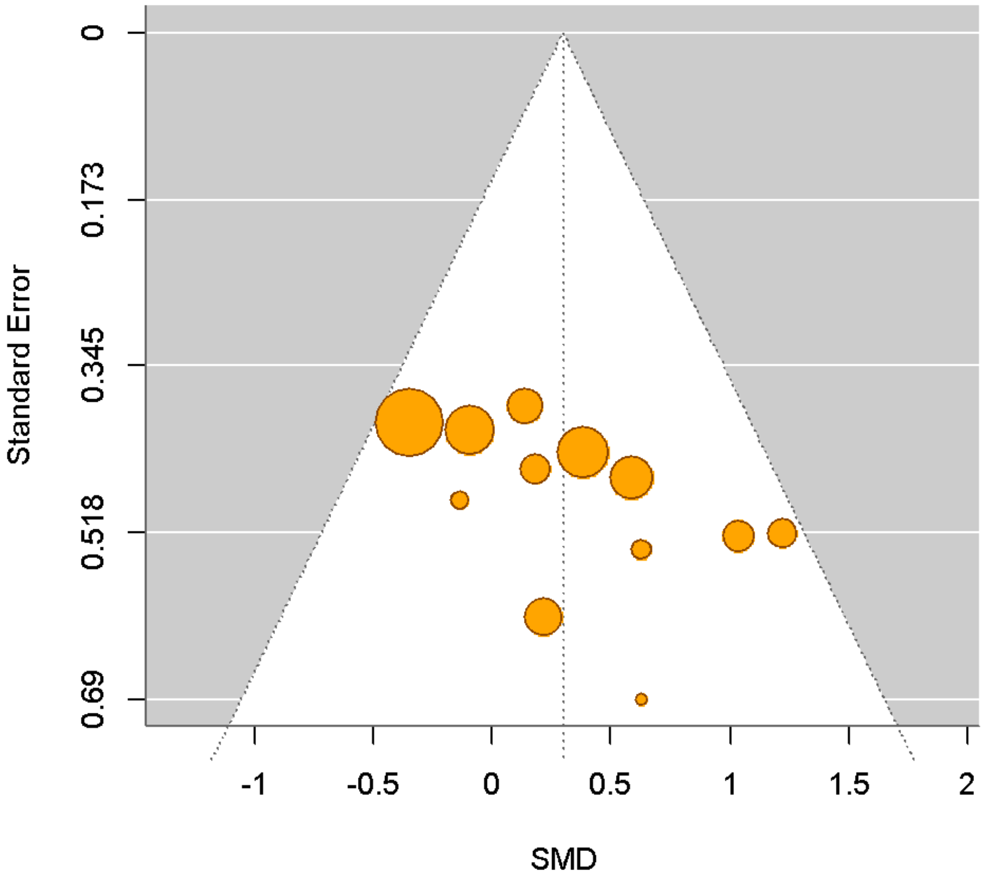

Notes.
Estimates were aggregated to the study level.
The plotted dots were weighted by the number of estimates per study.
SMD, standardized mean difference (i.e., Hedge's *g*).
In a multilevel meta-regression, the effect sizes' standard errors were included as the only moderator. The coefficient for the standard errors was not statistically significant (β = 4.14, 95% CI = -0.19 to 8.46, *p*-value = 0.061). Therefore, there was no evidence of asymmetry in the distribution of effect sizes.

© 2025 Crockett MA et al. *JAMA Network Open.*

eFigure 13. Funnel Plot of Short-Term Effects Between Active Interventions on Stigma-Related Behaviors

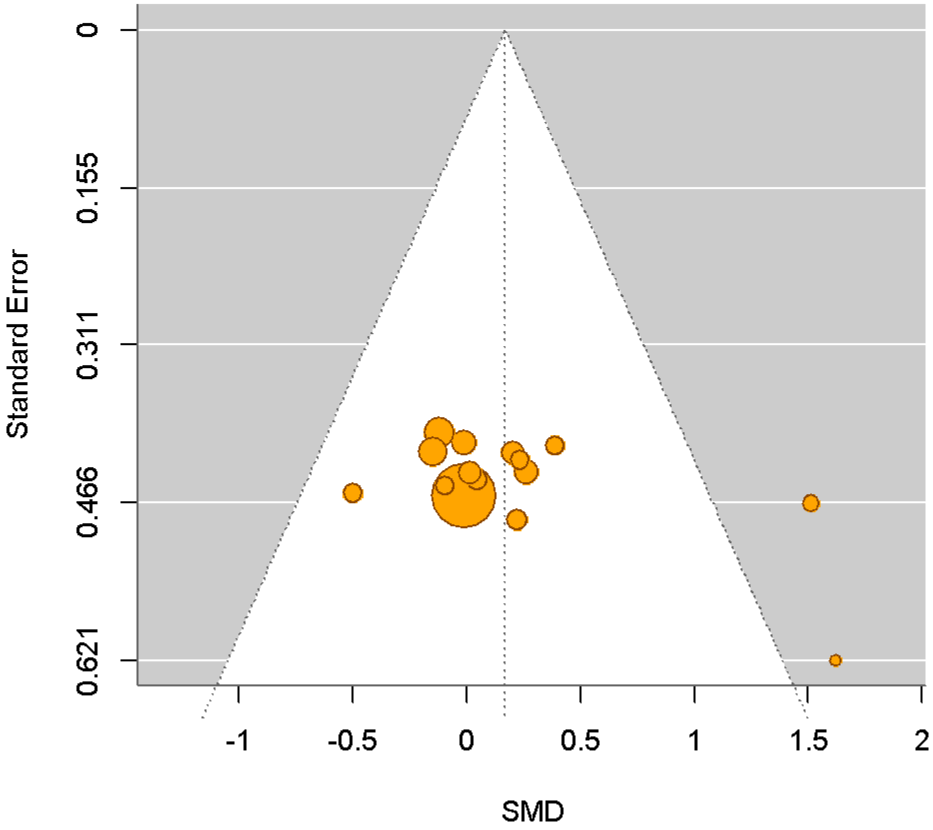

Notes.
Estimates were aggregated to the study level.
The plotted dots were weighted by the number of estimates per study.
SMD, standardized mean difference (i.e., Hedge's *g*).
In a multilevel meta-regression, the effect sizes' standard errors were included as the only moderator. The coefficient for the standard errors was statistically significant (β = 5.22, 95% CI = 1.95 to 8.49, *p*-value = 0.002). Therefore, there was evidence of asymmetry in the distribution of effect sizes.

© 2025 Crockett MA et al. *JAMA Network Open.*

eFigure 14. Funnel Plot of Short-Term Effects of Interventions vs Controls on Health-Seeking Intentions

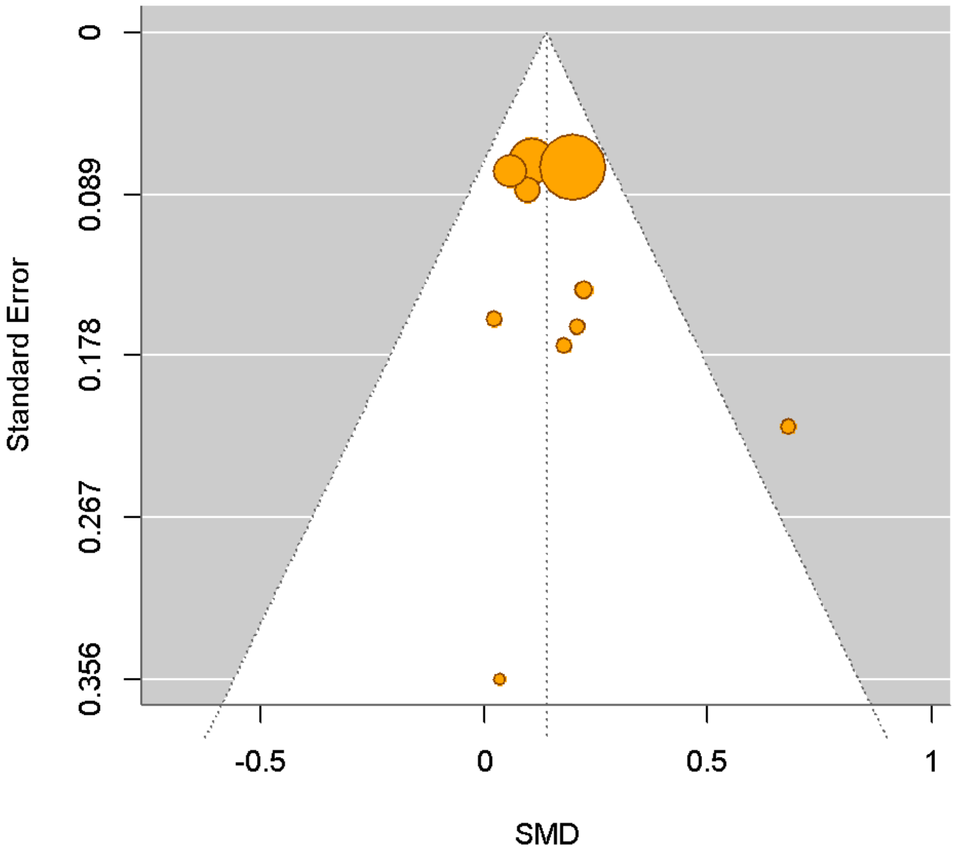

Notes.
Estimates were aggregated to the study level.
The plotted dots were weighted by the number of estimates per study.
SMD, standardized mean difference (i.e., Hedge's *g*).
In a multilevel meta-regression, the effect sizes' standard errors were included as the onl4. y moderator. The coefficient for the standard errors was not statistically significant (β = 0.18, 95% CI = -1.12 to 1.48, *p*-value = 0.780). Therefore, there was no evidence of asymmetry in the distribution of effect sizes.

© 2025 Crockett MA et al. *JAMA Network Open.*
