# Supplementary material for: Interventions to Reduce Mental Health Stigma in Young People: A Systematic Review and Meta-Analysis
Source: JAMA Netw Open. 2025 Jan 15;8(1):e2454730. doi: 10.1001/jamanetworkopen.2024.54730 (PMC11736514; doi:10.1001/jamanetworkopen.2024.54730)
Supplement: Supplement 2. — Data Sharing Statement [file jamanetwopen-e2454730-s002.pdf]

Data Sharing Statement

Crockett. Interventions to Reduce Mental Health Stigma in Young People. *JAMA Netw Open*. Published January 15, 2025. doi:10.1001/jamanetworkopen.2024.54730

Data
Data available: Yes
Data types: Data (not involving human participants), Data dictionary
How to access data: The data that support the findings of this study are available from the corresponding author upon reasonable request.
When available: With publication

Supporting Documents
Document types: Statistical/analytic code
How to access documents: The data that support the findings of this study are available from the corresponding author upon reasonable request.
When available: With publication

Additional Information
Who can access the data: Anyone requesting the data
Types of analyses: For any purpose
Mechanisms of data availability: Without investigator support
